# Persistence of *Coxiella burnetii*, the Agent of Q Fever, in Murine Adipose Tissue

**DOI:** 10.1371/journal.pone.0097503

**Published:** 2014-05-16

**Authors:** Yassina Bechah, Johanna Verneau, Amira Ben Amara, Abdoulaye O. Barry, Catherine Lépolard, Vincent Achard, Laurence Panicot-Dubois, Julien Textoris, Christian Capo, Eric Ghigo, Jean-Louis Mege

**Affiliations:** 1 Unité de Recherche sur les Maladies Infectieuses Transmissibles et Emergentes, Aix-Marseille Université, UMR CNRS 7278, IRD 198, INSERM U1095, Marseille, France; 2 Nutrition, Obésité et Risque Thrombotique, Aix-Marseille Université, UMR_S INSERM, Marseille, France; 3 Aix-Marseille Université UMR-S1076, Endothélium, Pathologies Vasculaires et Cibles Thérapeutiques, Marseille, France; University of São Paulo, Brazil

## Abstract

*Coxiella burnetii*, the agent of Q fever, is known to persist in humans and rodents but its cellular reservoir in hosts remains undetermined. We hypothesized that adipose tissue serves as a *C. burnetii* reservoir during bacterial latency. BALB/c and C57BL/6 mice were infected with *C. burnetii* by the intraperitoneal route or the intracheal route. Adipose tissue was tested for the presence of *C. burnetii* several months after infection. *C. burnetii* was detected in abdominal, inguinal and dorsal adipose tissue 4 months post-infection, when no bacteria were detected in blood, liver, lungs and spleen, regardless of the inoculation route and independently of mouse strain. The transfer of abdominal adipose tissue from convalescent BALB/c mice to naïve immunodeficient mice resulted in the infection of the recipient animals. It is likely that *C. burnetii* infects adipocytes *in vivo* because bacteria were found in adipocytes within adipose tissue and replicated within *in vitro*-differentiated adipocytes. In addition, *C. burnetii* induced a specific transcriptional program in *in-vivo* and *in vitro*-differentiated adipocytes, which was enriched in categories associated with inflammatory response, hormone response and cytoskeleton. These changes may account for bacterial replication in *in-vitro* and chronic infection *in-vivo*. Adipose tissue may be the reservoir in which *C. burnetii* persists for prolonged periods after apparent clinical cure. The mouse model of *C. burnetii* infection may be used to understand the relapses of Q fever and provide new perspectives to the follow-up of patients.

## Introduction

*Coxiella burnetii,* an obligate intracellular bacterium, is the causative agent of Q fever, a worldwide zoonosis [1]. Humans contract *C. burnetii* infection through aerosol route after contact with infected animals or via the digestive tract after ingestion of dairy products [2]. Primary infection is asymptomatic in 60% of cases and manifests as self-limited febrile illness, pneumonia and/or hepatitis in symptomatic patients [2]. Despite efficient treatment, relapses of Q fever have been reported in patients after acute Q fever [1]. These relapses may be related to reactivation documented in mice during repeated pregnancies [3], [4] or treated with radiations [5] and in guinea pigs treated with cortisone [6]. The infection may become chronic in immunocompromised patients, pregnant women or patients with valvulopathy, and its principal manifestation is endocarditis. The treatment based on the combination of doxycycline and chloroquine has markedly improved the prognosis of Q fever endocarditis [7]. The natural history of *C. burnetii* infection demonstrates that the bacterium persists in hosts for prolonged periods; the persistence has been documented in bone marrow and in peripheral blood mononuclear cells from patients who have been apparently cured of Q fever; however, only nucleic acid rather than viable bacteria have been detected in these sites [8], [9]. Nonetheless, these findings suggest that *C. burnetii* persists in patients cured from Q fever despite adapted immune response and efficient treatment regimen.

The adipose tissue (AT) may be a preferentially infected by *C. burnetii*. AT not only acts as a storage depot for excess calories but also synthetizes fatty acids and adipokines that have systemic effects [10], [11]. In homeostatic conditions, AT is indirectly affected by commensal gut bacteria via the modulation of inflammatory response [12]. During the last decade, it has demonstrated that pathogens target AT. Indeed, *Listeria monocytogenes* is present in AT five days after infection of mice [13]. HIV may target human AT because AT expresses receptors for HIV, such as CD4, CCR5 and CXCR4 [14], [15]. AT has also emerged as a reservoir for different pathogens. *Trypanosoma cruzi*, the agent of Chagas' disease, also targets the AT of infected mice and its DNA remains detectable for at least 300 days after the primary infection [16], [17]. *Mycobacterium tuberculosis* also uses human AT as a reservoir [18], [19]. We recently showed that *Rickettsia prowazekii*, the agent of epidemic typhus, is detected in AT of mice four months after clinical cure of the primary infection, when rickettsiae were absent from other organs including liver, lungs, brain and spleen [20].

In this work, we show that *C. burnetii* persisted in AT of BALB/c and C57BL/6 mice for at least four months after infection and the transfer of abdominal adipose tissue from convalescent BALB/c mice to naïve immunodeficient mice resulted in the infection of the recipient animals. We also show that *C. burnetii* infected cultured adipocytes in which bacteria induced a specific gene program expression. The presence of *C. burnetii* in AT may explain bacterial persistence and clinical complications in Q fever and open new perspectives in the follow-up of patients.

## Materials and Methods

### *Ethics statement*

The experimental protocol was approved by the Institutional Animal Care and Use Committee of the Aix-Marseille Université, France. Mice were handled according to the rules of Décret N° 8 87–848, October 19, 1987, Paris. Animals housed in a specific facility and fed sterile food and water ad libitum were observed twice per day for any signs of discomfort or distress.

Intra-tracheal (IT) inoculation was performed under ketamin/xylazine anesthesia and all efforts were made to minimize suffering.

### *Bacterial preparation*

*Coxiella burnetii* (RSA493 Nine Mile strain) was cultured in L929 cells. Cell monolayers were infected for 7 to 10 days after which bacteria were harvested, sonicated and quantified as previously reported [21]. Bacterial viability was assessed using the LIVE/DEAD BacLight kit (Molecular Probes).

### *Mice infection and tissue sampling*

Seven week-old female BALB/c, C57BL/6 and nude mice were obtained from Charles River laboratories. Female CX3CR1^GFP^ C57BL/6 transgenic mice with GFP-labeled immune cells, including macrophages and natural kills cells purchased from Jackson Laboratories were also used in some experiments [22]. Mice were inoculated with 10^6^ bacteria using the intra-peritoneal route (IP). In a different series of experiments, one group of BALB/c mice was inoculated using IT route, as previously reported [23]. At days 1, 8, 15, 30, 60, 90 and 120 post-infection (p.i.), 200 µl of whole blood from mice were collected by retro-orbital puncture and mice (n = 3 infected mice and n = 2 uninfected mice) were sacrificed. About 25 mg of AT from abdominal, inguinal and dorsal regions and 25 mg samples from liver, lungs and spleen were collected and conserved at −80°C for quantitative real-time PCR (qPCR) and quantitative real-time reverse transcription PCR (qRT-PCR) assays and cell culture. Samples of AT were also fixed in 5% formalin for immunostaining assays. Samples of abdominal adipose tissue from infected BALB/c mice were taken, homogenized and transfered to naive nude mice via IP route.

### *Adipocyte differentiation and infection*

Murine adipocytes were obtained using two different methods. First, the cells from the fibroblast cell line 3T3-L1 were differentiated into adipocytes, as previously reported [20]. Briefly, cells seeded at 4×10^4^ cells/well were incubated in Dulbecco's modified Eagle's medium (DMEM) (Life Technologies) with 10% fetal calf serum (FCS) and 2 mM L-glutamine until confluence. Adipocyte differentiation was obtained by adding 100 mM 3-isobutyl-1-methylxanthine (IBMX), 250 nM dexamethasone and 1 µg/ml insulin (Sigma-Aldrich). After 2 days, the medium was replaced by DMEM containing 10% FCS and 1 µg/ml insulin and was changed every 2 days. Second, visceral fat depots from BALB/c mice were sampled and stromal vascular cells containing pre-adipocytes cells were isolated in 10% collagenase (Sigma-Aldrich) for 1 hour at 37°C, as previously described [24]. Cell suspensions were filtered and centrifuged at 600×*g* for 5 min. The obtained pellets were cultured in 24-well plates for 24 hours at 37°C. Pre-adipocytes were differentiated into adipocytes by adding the adipogenic hormone combination. By a 2 week-culture, the adipocyte differentiation was checked by red oil and bodipy 493/503 staining of lipid droplets. Adipocyte infection was performed as follows. Cells were incubated with *C. burnetii* for 4 hours, extensively washed to discard unbound pathogens (time designated as day 0) and incubated in DMEM containing 10% FCS for different periods of time at 37°C.

### *Determination of C. burnetii infection*

Four complementary methods were used to analyze *C. burnetii* infection of AT and adipocytes. The presence of *C. burnetii* in the blood (200 µl) and tissues (25 mg) from mice and adipocytes was assessed using qPCR, as previously reported [23]. DNA was extracted using a QIAamp Tissue Kit (Qiagen) in 100 µl final volume. qPCR was performed using the LightCycler system (Roche Diagnostics) with 5 µl DNA extract and specific primers and probe targeting a fragment of the *C. burnetii* 16S DNA gene. The selected primers and probe specific for *C. burnetii* were F (5′-ACGGGTGAGTAATGCGTAGG-3′); R (5′-GCTGATCGTCCTCTCA-GACC-3') and probe P (6-FAM-GCAAAGCGGGGGATCTTCGG-TAMR). Negative controls consisted of DNA extracted from blood and tissues from uninfected mice or from uninfected adipocytes. Each PCR run included a standard curve of 10-fold serial dilutions of a known concentration of *C. burnetii* DNA. The infection of AT was also studied by immunostaining, as previously described [20]. Briefly, 5 µm paraffin-embedded sections of AT were obtained and the presence of *C. burnetii* was determined using a rabbit polyclonal antibodies (Abs) directed against *C. burnetii* (1∶500 dilution). Bacteria were revealed using Alexa 488-conjugated F(ab') anti-rabbit IgG for immunofluorescence studies or biotin-conjugated Abs and peroxidase-labeled streptavidin for immunohistochemistry studies. The viability of *C. burnetii* in AT was evaluated by culturing dissociated AT on confluent monolayers of L929 cells, known to support *C. burnetii* replication, as previously described [20]. The presence of bacteria within L929 cells was revealed using immunofluorescence. The infection of adipocytes cultured on glass coverslips was finally evaluated by immunofluorescence [20]. After fixation with 3% paraformaldehyde and permeabilization with 0.1% Triton X-100, adipocytes were incubated with rabbit Abs directed against *C. burnetii* (1∶500 dilution) for 30 min and then with Alexa 488-conjugated F(ab') anti-rabbit IgG (Molecular Probes) (1∶500 dilution). Nuclei and lipid droplets were counterstained with DAPI and bodipy 493/503 (Life Technologies), respectively. Images were analyzed with confocal microscope (see below).

### *Intracellular localization of C. burnetii*

The compartment in which *C. burnetii* was localized was determined by confocal microscopy. After fixation and permeabilization, 3T3-L1-differentiated adipocytes were incubated with mouse Abs specific for lysosomal-associated membrane protein (Lamp)-1, a marker of late endosomes and lysosomes (1∶500 dilution, Abcam), rabbit Abs specific for cathepsin D, a lysosomal protease, (1∶1000 dilution, a gift from Dr. Kornfeld, St. Louis, MO) and human Abs directed against *C. burnetii* (1∶500 dilution) obtained from patients with Q fever [21]. After washing, adipocytes were incubated with secondary fluorescent Abs (Life Technologies), washed, mounted with Mowiol and analyzed using epifluorescence and laser scanning confocal microscopy. Images were acquired using a confocal microscope (Leica TCS SP5) with a 63X/1.32-0.6 oil objective, an electronic magnification of 1.5× and a resolution of 1024×1024 pixels.

### *Microarrays and data analysis*

3T3-L1-differentiated adipocytes were stimulated with live *C. burnetii* or heat killed bacteria (50 bacteria per cell) for 8 hours. Total RNA was extracted using an RNeasy Mini Kit (Qiagen) and DNase treatment. The integrity and the amount of RNA were assessed and microarray studies were performed according Agilent Technologies, as recently described [21]. Briefly, four samples per experimental condition (stimulated and not stimulated) were used. Labeled cRNAs were obtained from 300 ng of RNA using T7 RNA polymerase and cyanine 3-labeled CTP (Cy-3) fluorescent dyes with One-Color Microarray-Based Gene Expression Analysis (Agilent Technologies). Labeled cRNAs were hybridized onto Whole Mouse Genome Oligo Microarrays 4×44k kit (G4852A), representing 44,000 60-mer oligonucleotides, for 17 hours at 65°C. Slides were scanned at a 5-µm resolution with a G2505B DNA microarray scanner (Agilent Technologies). Image analysis and intra-array signal correction were performed using Agilent Feature Extractor Software A.9.1.3. Supervised analyses were carried out with R (R for statistical computation and graphic version 2.15) and the BioConductor library [25]. False Discovery Rate (FDR) was set to 0.05 to filter modulated genes. The selected probes were further filtered for differentially expressed genes using an absolute fold change (FC) ≥1.5. Functional annotation was performed using the DAVID Bioinformatics Resource 2008 [26], [27]. The gene networks were generated using the PathwayStudioTM software (Ariadne Genomics). The data have been submitted to the NCBI Gene Expression Omnibus (GEO) and are accessible through GEO Series accession number GSE46335.

### *Quantitative real-time reverse transcription PCR (qRT-PCR)*

The cDNA was prepared using 5 ng of total RNA, oligo(dT) primer and M-MLV reverse transciptase, as previously described [28]. Reverse transcriptase was omitted in negative controls. PCR was performed using the LightCycler system with SYBR Green PCR Master Mix (Roche Diagnostics), 2 µl of the prepared cDNA and specific primers. The used primers are listed in **Table S1**. The results were normalized to the expression of β-actin and the fold change (FC) was calculated as follows: FC = 2−^ΔΔct^, where ΔΔct =  (Ct_target_ - Ct_actin_)_stimulated_ - (Ct_target_ - Ct_actin_)_unstimulated_ [28]. The expression of genes was considered as modulated when FC≥2.0.

### *Statistical analysis*

The results of DNA copies are expressed as the means ±SEM and were compared using an unpaired t test. Statistical analyses were performed using GraphPad-Prism software (Version 5.0). The data were considered as significant when the *P* value <0.05.

## Results

### *C. burnetii persistence in AT*

**Figure 1:**
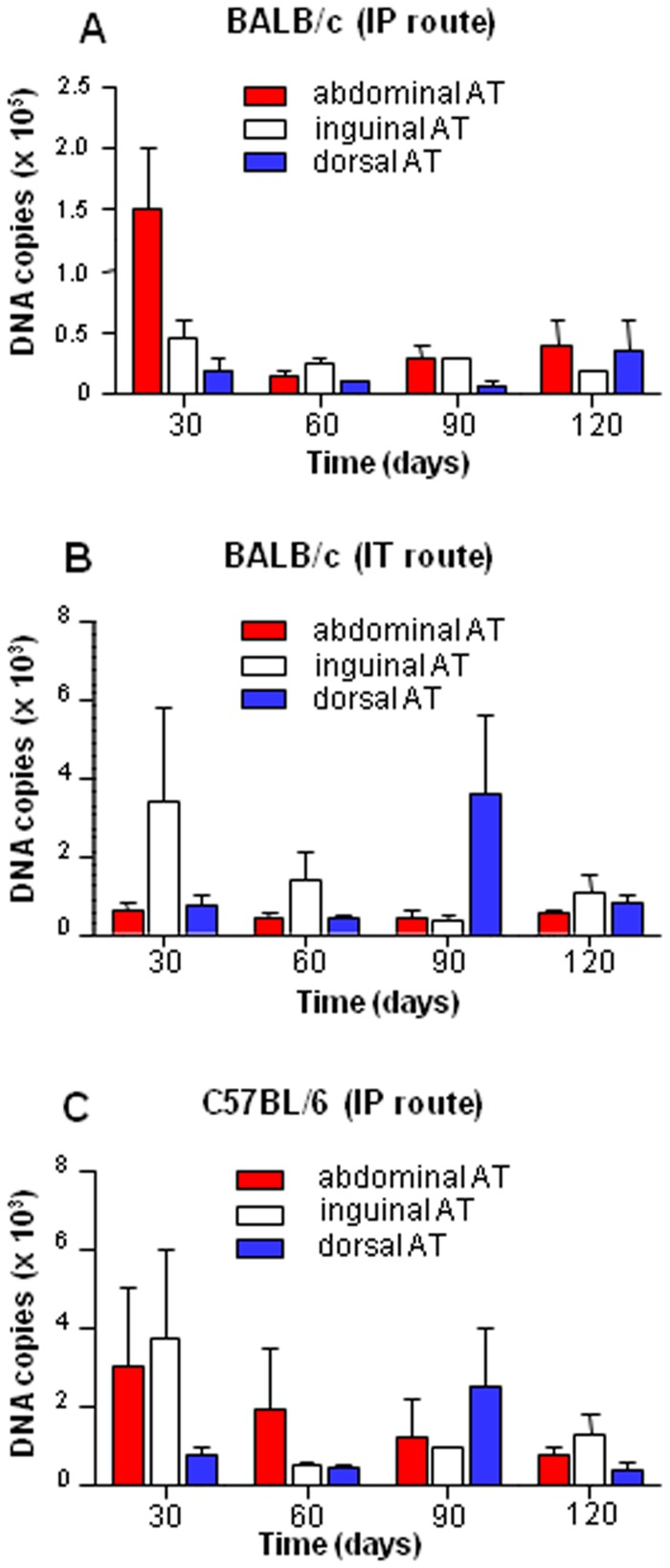
***C. burnetii*** persistence in AT from mice. Abdominal, inguinal, and dorsal (25 mg) AT from mice inoculated with *C. burnetii* were collected after different periods of time, DNA was extracted in a 100 µl volume and the presence of *C. burnetii* DNA was determined by qPCR using a 5 µl DNA extract. AT from BALB/c mice inoculated with *C. burnetii* via IP route (**A**), from BALB/c mice infected via the IT (**B**) route and from C57BL/6 mice infected via the IP route (**C**) were sampled. Results represented the number of DNA copies for total sample (25 mg) and were expressed as mean ±SEM.

*C. burnetii* was inoculated to BALB/c mice by the IP route. Mice were sacrificed after different periods of time and tissue samples were analyzed for the presence of *C. burnetii*. At 30 days p.i., no bacterial DNA copies were found in blood, spleen, liver and lungs. In contrast, they were detected in abdominal AT (about 2×10^5^ copies) and were still present after 120 days (about 3×10^4^ copies) (**Figure 1A**). As the bacterial material found in the abdominal region may be associated to the local injection of *C. burnetii*, we studied the infection of inguinal and dorsal AT. The inguinal AT was infected and the infection was persistent until 120 days p.i. (about 1.5×10^4^ copies) (**Figure 1A**). Similarly, bacterial DNA copies were detected in dorsal AT after 30 days p. i. (about 2.5×10^4^ DNA copies) and persisted at 120 days p.i. (**Figure 1A**). The infection burden was similar in the three locations of AT (abdominal, inguinal and dorsal) since the differences in DNA copies were not significant for all time points.

Because the presence of *C. burnetii* in AT may be associated with the inoculation route, we infected BALB/c mice using the IT route. At day 30 p.i., while no bacterial DNA was detected in blood, *C. burnetii* DNA was found in abdominal, inguinal and dorsal AT although the IT route was less efficient than the IP route, but no statistical significant difference between the IP and the IT routes was demonstrated. The DNA copies were still detected after 120 days p.i. (**Figure 1B**), demonstrating the persistence of bacterial material within the AT.

The genetic background of mice may also affect the persistence of *C. burnetii* within AT. C57BL/6 mice were inoculated with *C. burnetii* using the IP route. After day 30 p.i. bacterial DNA was not found in blood, liver, lung and spleen of C57BL/6 mice but was detected in abdominal, inguinal and dorsal AT. *C. burnetii* DNA persisted for at least 120 days in AT from C57BL/6 mice (**Figure 1C**) and no statistical significant difference was observed between BALB/c and C57BL/6. Hence, *C. burnetii* infection was not dependent on the genetic background of mice.

**Figure 2:**
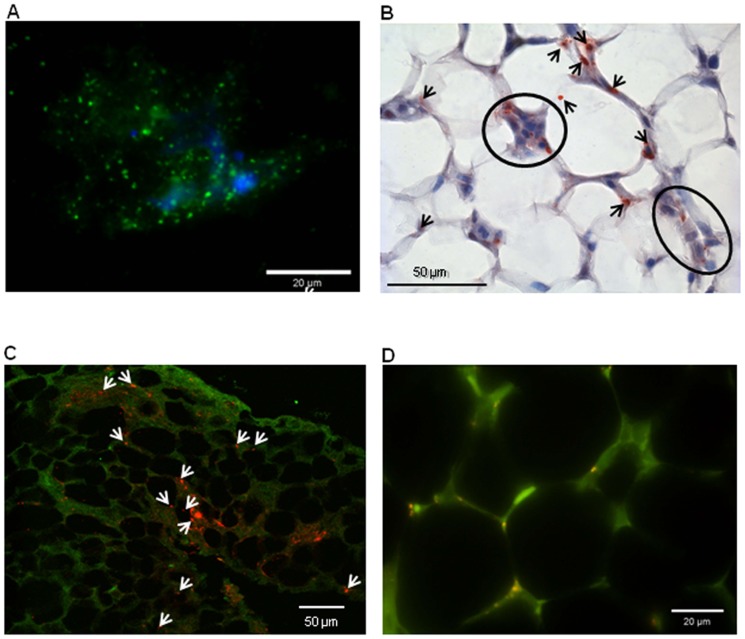
Cell culture and immunostaining of ***C. burnetii*** within adipose tissue. **A**, *C. burnetii* was detected in dilacerated inguinal AT at day 60 p.i. incubated with L929 cells after 40 days of culture. Bacteria were detected using specific antibodies conjugated with Alexa 488 (in green). Original magnification: X100. **B**, The presence of *C. burnetii* was determined in sections of paraffin-embedded abdominal AT at day 30 p.i. using a rabbit anti-*C. burnetii* polyclonal antibodies. Bacteria were revealed using biotin-conjugated antibodies and peroxidase-labeled streptavidin. Labeled bacteria appear in red in the inflammatory infiltrate (circle) and in adipocytes (arrows). Original magnification: X400. **C**, Immunodetection of *C. burnetii* in sections of paraffin-embedded abdominal AT at day 30 p.i. Bacteria were revealed using rabbit anti-*C. burnetii* polyclonal antibodies and Alexa 555-conjugted anti-rabbit IgG (red). Labeled bacteria in red (arrows) were observed in AT (green, artificially attributed color) using confocal microscopy. **D**, Sections of paraffin-embedded abdominal AT from C57BL/6 transgenic mice with GFP-labeled immune cells inoculated with *C. burnetii* via the IP route at day 10 p.i. were incubated with rabbit anti-*C. burnetii* polyclonal antibodies. Bacteria were revealed using Alexa 555-conjugated F(ab') anti-rabbit IgG. Bacteria appear in red in the macrophages and in adipocytes. Original magnification: X100.

The presence of *C. burnetii* in the AT of BALB/c mice was confirmed using cell culture. The inguinal AT sampled at 60 days p.i. was homogenized and inoculated to L929 cells. After 40 days of culture, *C. burnetii* was detected by immunofluorescence within L929 cells (**Figure 2A**). Live bacteria were also isolated from abdominal and dorsal AT sampled between 30 and 120 days, but not from liver, lung or spleen that are revealed negative by PCR.

We next studied the presence of *C. burnetii* within the AT using immunohistochemistry and immunofluorescence. Interestingly, bacteria were detected in AT, and the anti-*Coxiella* staining appears to be associated with the inflammatory infiltrate as well as with adipocytes (**Figure 2B**). Using immunofluorescence, *C. burnetii* was also found in adipocytes and the staining of bacteria suggests an intracellular location (**Figure 2C**). Using mice with GFP-labeled immune cells including macrophages and natural killer cells, we found that *C. burnetii* targeted adipocytes and likely macrophages from abdominal AT (**Figure 2D**).

Finally,_samples of abdominal AT from infected BALB/c mice were taken at day 15 p.i. when no bacteria were detected in blood and transfered to naïve nude mice. This transfer of AT from convalescent BALB/c mice to naïve, immunodeficient mice resulted in infection of the recipient animals as demonstrated by qPCR. Indeed, at day 4 post-transfer, the nude mice were euthanized and the infection was demonstrated in the blood of 2/4 mice, the spleen of 2/4 mice and the brain of 2/4 mice. In addition, the abdominal AT of 2/4 nude mice was also_positive (**Table 1**).

**Table 1:** Mean DNA copies of *C. burnetii* in the organs of nude mice transplanted with BALB/c-infected abdominal adipose tissue.

Mean DNA copies/200 µl blood or 25 mg tissue (minimum and maximum values)		
Samples		no. mice positive/no. tested mice
**Blood**	2.50×10^3^(2.4×10^3^–2.5×10^3^)	2/4
**Spleen** *	1.75×10^3^(1.8×10^3^–1.7×10^3^)	2/4
**Brain**	7.93×10^4^(1.5×10^5^–1.0×10^4^)	2/4
**Abdominal AT**	5.69×10^4^(9.8×10^4^–1.6×10^4^)	2/4

*DNA was extracted from 10 mg of tissue.

AT: adipose tissue.

Collectively, our results show that *C. burnetii* persists in the AT of mice for prolonged periods, and provides a potential reservoir for *C. burnetii* persistence.

### *C. burnetii infection of adipocytes*

**Figure 3:**
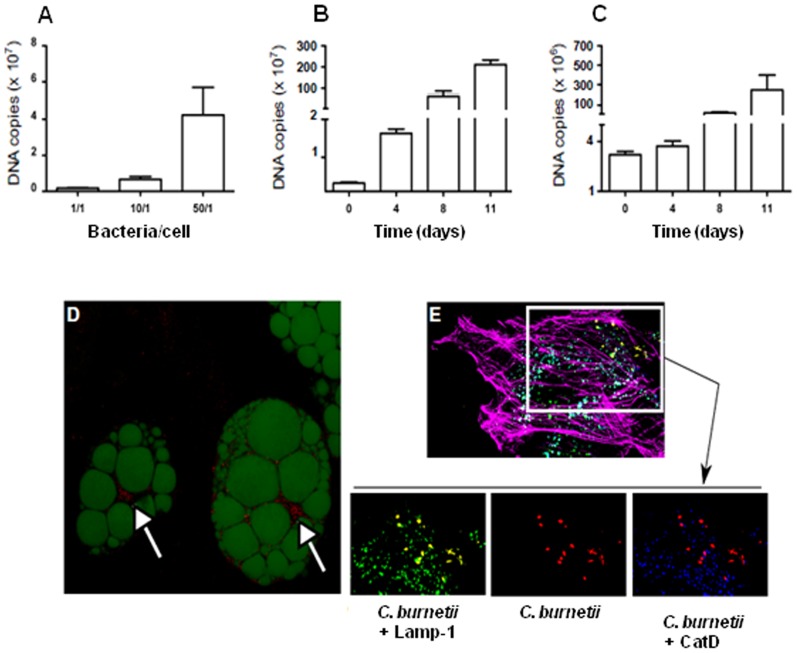
Intracellular fate of ***C. burnetii*** in adipocytes. **A-C**, Adipocytes differentiated from the fibroblast cell line 3T3-L1 were incubated with increasing doses of *C. burnetii* (A). Adipocytes differentiated from the fibroblast cell line 3T3-L1 (B) or stromal vascular cells (C) were incubated with 10 bacteria per cell for 4 hours. After washing (defined as day 0), adipocytes were incubated at 37°C for different periods of time. Bacteria were detected using qPCR and the results are represented as the mean values ±SEM (n = 8). **D**, Bacteria and lipid droplets were labeled with human anti-*C. burnetii* antibodies and bodipy 493/503, respectively. A representative micrographs obtained by confocal microscopy is shown. Bacteria are displayed as red, and lipid droplets are displayed as green. E, 3T3-L1-differentiated adipocytes were incubated with 10 *C. burnetii* per cell for 4 hours, washed to remove unbounded bacteria, and cultured for 8 days. Infected cells were labeled with anti-*C. burnetii* (Alexa 555), anti-Lamp-1 (Alexa 488), anti-cathepsin D (CatD) (Alexa 647) antibodies and bodipy phallacidin (to label filamentous actin) and were analyzed by laser scanning confocal microscopy. The co-localization of bacteria (red) with Lamp-1 (green) and cathepsin D (blue) is shown by merging the respective fluorescent images. *C. burnetii* co-localized only with Lamp-1 marker (yellow).

Because *C. burnetii* infects adipocytes *in vivo*, we used adipocytes differentiated from fibroblastic 3T3-L1 cells to analyze their infection. *C. burnetii* was ingested in a dose-dependent manner (**Figure 3A**) and replicated within adipocytes, as determined by qPCR. Indeed, using a dose of 10 bacteria per cell, we studied the intracellular fate of *C. burnetii*. The number of bacterial DNA copies significantly increased at day 4 p.i. (*P* = 0.0001) and at days 8 and 11 p.i. (*P* = 0.004 and *P* = 0.0001, respectively) as compared to day 0 (**Figure 3B**). The same pattern of replication was observed with adipocytes differentiated from pre-adipocytes isolated from stromal vascular cells. The number of bacterial DNA copies did not significantly increase at day 4 p.i., but they dramatically increased in these adipocytes at days 8 and 11 p.i., as determined by qPCR (*P* = 0.01 and *P* = 0.009, respectively) as compared to day 0 (**Figure 3C**). Bacteria were also observed within adipocytes using confocal microscopy (**Figure 3D**). The compartment in which *C. burnetii* resides in adipocytes was defined via immunodection of compartment-specific markers. *C. burnetii* co-localized with Lamp-1 but did not co-localize with cathepsin D, indicating that *C. burnetii* resided within late phagosomes in adipocytes (**Figure 3E**). Taken together, our results show that *C. burnetii* is able to infect and replicate within adipocytes.

### *Transcriptional profile of infected adipocytes*

**Figure 4:**
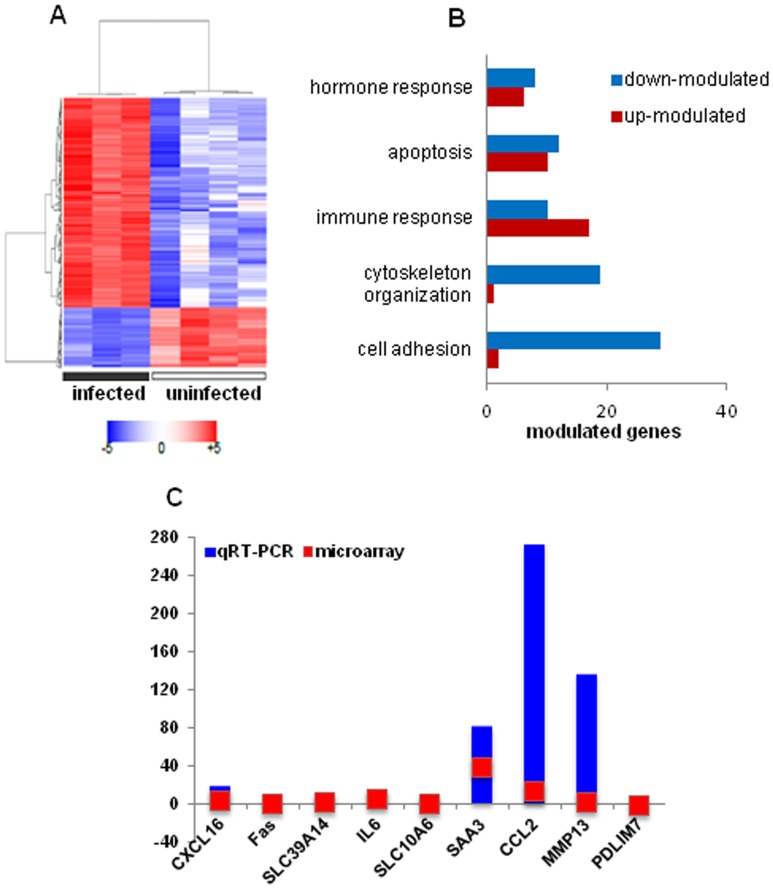
Microarray analysis of adipocytes stimulated by ***C. burnetii***. Adipocytes differentiated from the fibroblast cell line 3T3-L1 were incubated with 50 bacteria per cell for 8 hours. **A**, Heatmap representation of modulated genes selected by supervised analysis in stimulated and unstimulated adipocytes. Genes (in rows) and samples (in columns) were organized by hierarchical clustering (Pearson correlation distance, average linkage). Gene expression levels are color coded from blue (down-modulated) to red (up-modulated). **B-C**, Functional annotation of modulated genes and qRT-PCR results. The modulated genes in the most enriched GO terms are shown (B). qRT-PCR confirmation of some genes of the most enriched GO terms identified by microarray analysis (C). qRT-PCR ratios are presented as bleu bars and microarray ratios are presented as red bars. Both methods gave similar results (Spearman correlation coefficient  = 0.85, *P* value  = 0.006).

**Figure 5:**
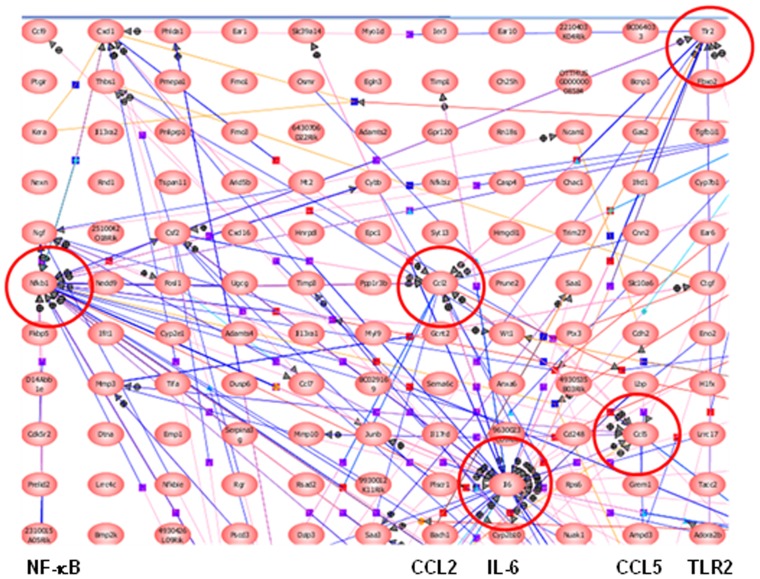
Networks induced in ***C. burnetii***-stimulated adipocytes. The pathways induced in cultured adipocytes by *C. burnetii* stimulation were generated using the PathwayStudioTM software. Note that networks were organized around *IL6*, *TNF*, *CCL2*, *CCL5*, *NF-kB* and *TLR2* genes.

To study the 3T3-differentiated adipocytes responses to *C. burnetii*, we tested different kinetics of stimulations (2, 4, 8 and 24 hours) and we selected 8 hours for the expression of three adipokines (TNF, IL-6, and CCL2) that was highest at this time point as assessed by qRT-PCR (**Figure S1**). Using microarray, we found that the interaction of *C. burnetii* with adipocytes induced a specific transcriptional signature. Microarray analysis showed that 600 probes (466 genes) were significantly modulated (FC≥1.5 and FDR<0.05) after an 8-hour stimulation: they consisted of 132 up-modulated probes (FCs ranging from 1.5 to 106) and 468 down-modulated probes (FCs ranging from −5 to −1.5) (**Figure 4A**; the complete list of modulated probes is provided in **Table S2**. The analysis of modulated genes using Gene Ontology (GO) terms revealed enrichment for genes implicated in “cell adhesion”, “cytoskeleton organization”, “hormone response” “apoptosis” and “immune response” (**Figure 4B**). In these enriched GO terms, most of genes included in “cell adhesion” (29 out of 31 genes) and “cytoskeleton organization” (19 out of 20 genes) were down-modulated. By contrast, approximately one half of the genes included in “hormone response” (6 out of 14 genes), “apoptosis” (10 out of 22 genes) and “immune response” (17 out of 27 genes) GO terms were up-modulated (**Figure 4B**). We selected a sampling of genes (*CXCL16*, *Fas*, *SLC39A14*, *IL6*, *SLC10A6*, *SAA3*, *CCL2*, *MMP13* and *PDLIM7*) that we found to be modulated in microarray experiments and tested their modulation using qRT-PCR. Microarray and qRT-PCR ratios were highly correlated (Spearman correlation coefficient  = 0.85, *P* value  = 0.006) (**Figure 4C**). We tested also whether modulated genes belong to specific gene networks. We found that *C. burnetii* induced gene networks around two inflammatory cytokines (IL-6, TNF), two chemokines (CCL2, CCL5), the transcription factor NF-kB and TLR2, known to be involved in the inflammatory response (**Figure 5**). Finally, we measured the expression of a set of genes in response to heat killed bacteria, which was measured in response to live bacteria. The heat-killed bacteria induced this set of genes but the expression level was lower than that of live bacteria (**Figure S2**), demonstrating that the response seen in adipocytes is partly dependent on bacterial virulence. Taken together, these results suggest that *C. burnetii* stimulation of adipocytes results in an intense transcriptional inflammatory response.

### *Gene expression in infected adipose tissue*

We wondered if the genes modulated in *C. burnetii*-infected adipocytes were modulated in AT after *in vivo* infection. To address this issue, we_examined the expression of set of genes in AT from infected mice using qRT-PCR. During the acute *C. burnetii*-infection (4 and 8 days p.i.) the expression of several inflammatory genes was up-regulated to different degrees in abdominal, inguinal and dorsal AT (**Figure S3**). The expression of *IL6* was down-modulated only in abdominal AT at day 4 p.i., and down-modulated in abdominal and dorsal AT at day 8 p.i.. Interestingly, the up-regulation of inflammatory markers such as *CCL2*, *CXCL16*, *Fas*, *SAA3* and *SLC39A14* persisted even at day 15 p.i. in abdominal and/or dorsal AT (**Figure S3A,C**). At day 30 p.i., we could not detect any modulation in gene expression, except for *Fas* which was up-regulated in abdominal, inguinal and dorsal AT and *SLC10A6* which was up-regulated in abdominal AT (**Figure S3**). Note that the expression of *PDLIM7* was not detected in the 3 tested AT and for all tested time points. The transcriptional changes in AT suggested that *C. burnetii* affects the local inflammatory state within AT.

## Discussion

To understand *C. burnetii* persistence in humans despite treatment, we searched for a tissue reservoir for this organism. Because AT has been demonstrated to be targeted and/or be a reservoir for several intracellular pathogens able to relapse [13]-[16], [18], [20], we hypothesized that AT could also be a reservoir for *C. burnetii*. We found *C. burnetii* in abdominal, inguinal and dorsal AT from BALB/c and C57BL/6 mice for at least 4 months p.i., demonstrating that *C. burnetii* persisted in AT independently of the site of inoculation and the genetic background of mice. Interestingly, *C. burnetii* material was not detected in the blood, spleen, liver or lungs as early as 30 days of infection, suggesting that AT may serve as *C. burnetii* reservoir.

The presence of *C. burnetii* in the white (abdominal and inguinal) and the brown (dorsal) AT indicated that *C. burnetii* targeted the AT independently of their physiological role. Indeed, it is well known that white AT is involved in energy storage whereas brown AT plays a major role in thermogenesis regulation [29]. It has been also demonstrated that the visceral AT such as abdominal AT exerts metabolic and inflammatory activities higher than the subcutaneous AT (inguinal AT) [11]. As AT contains essentially adipocytes but also other types such as macrophages [10], we studied the localization of *C. burnetii* within AT cells by immunohistochemistry. Bacteria were detected in inflammatory infiltrates and in adipocytes (**Figure 2B**). Using mice with GFP-labeled immune cells including macrophages and natural killer cells, we also found that *C. burnetii* targeted macrophages and likely adipocytes (**Figure 2D**). To our knowledge, the nature of AT cells in which HIV, *T. cruzi*, *M. tuberculosis*, *L. monocytogenes* and *R. prowazekii* reside has not been investigated.

Because *C. burnetii* clearly infected adipocytes within AT, we studied the ability of *C. burnetii* to infect cultured adipocytes using two models of adipocytes, adipocytes differentiated from 3T3 cells and adipocytes differentiated from pre-adipocytes isolated from stromal vascular cells. *C. burnetii* replicated similarly in each type of adipocytes. It has been demonstrated that *HIV*, *R. prowazekii, T. cruzi* and *L. monocytogenes* [13]–[17], [20] replicate within cultured adipocytes differentiated from 3T3 L-1 cells. In contrast, *M. tuberculosis* and *Staphylococcus aureus* persist but does not replicate within these adipocytes [18], [30]. We also found that *C. burnetii* resided in cultured adipocytes in late phagosomes unable to fuse with lysosomes. This is reminiscent of the compartment in which *C. burnetii* resides in macrophages [31]. Again, to our knowledge, the intracellular compartment in which pathogens reside in cultured adipocytes is not known. The mechanism used by *C. burnetii* to inhibit the fusion of late phagosomes with lysosomes in macrophages has recently been elucidated. Through its lipopolysaccharide, *C. burnetii* inhibits the activation of a molecular complex composed of Vacuolar protein storing 41 (Vps41), homotypic protein storing (HOPS) and p38α mitogen-activated protein kinase (p38α-MAPK) required for trafficking bacteria to phagolysosomes [32]. We suggest that a similar mechanism operates in adipocytes.

Although *C. burnetii* efficiently replicated within cultured adipocytes, no bacterial replication was observed in AT. This difference could be related to the crosstalk of adipocytes with other AT cells including macrophages that were potentially targeted by *C. burnetii*. The restrained bacterial replication in AT may be related to the reduced availability of oxygen in AT that may inhibit *C. burnetii* growth [33]. AT is a source of inflammatory molecules cytokines, chemokines, growth factors and hormones such as leptin, a key regulator of inflammation [34], [35]. The presence of these molecules may also explain differences between *in vivo* and *in vitro* data. Regardless of the mechanisms that limit *C. burnetii* replication in the AT, its survival for prolonged periods may favor the establishment of a latent infection and may represent the reservoir from which relapse can initiate. This suggestion was illustrated by the fact that the infection can be induced in naïve mice by transfer of AT from convalescent infected animals.

*C. burnetii* clearly induced a transcriptional inflammatory profile in adipocytes, as demonstrated by gene networks around *CCL2*, *CCL5*, *IL6*, *TNF*, *NF-κB* and *TTR2*. It has been also found that cultured adipocytes release CCL2 and IL-6 when they were infected by *S. aureus* [30], *L. monocytogenes* [13] or *T. cruzi* [36]. As these pathogens infect adipocytes, we hypothesize that the inflammatory response observed in adipocytes may be associated with the latent infection of AT. In addition, IL-6 induces lipodystrophy [37] that likely favors the release of pathogens from AT into the circulation and, as a consequence, the induction of relapses. *C. burnetii* infection also induced the expression of the inflammatory genes within AT. This could be involved in the establishment of a chronic infection.

The long-term persistence of *C. burnetii* in AT may account of the reactivation of *C. burnetii* infection reported in mice treated with corticoids [6]. More recently, we found that the primary infection with *R. prowazekii* is reactivated in mice under dexamethasone treatment [20]. As corticoids are well known to depress the inflammatory response [38] and to induce lipodystrophy through the decreased expression of lipoprotein lipase, an enzyme secreted by adipocytes and involved in triglyceride synthesis [39], we hypothesize that the defective immune response and the lipodystrophy induced under corticotherapy may favor the release of pathogens from AT and, consequently, the reactivation of infection. In humans, relapses of *C. burnetii* infection are observed in immunocompromised patients (pregnant women, patients with cancer or under corticotherapy) [1], [2]. We may suppose that Q fever relapses occur via a similar mechanism, involving AT as reservoir and depressed immune response and lipodystrophy as triggering.

In conclusion, our findings could have potential clinical implications. Despite long term antibiotic therapy, relapses are relatively frequent in chronic Q fever [40], [41]. In this context, it is possible that antibiotics are often ineffective against *C. burnetii* because of poor penetration of AT. Structural changes in drug design may improve the penetration of AT by antibiotics active against *C. burnetii* and possibly others that persist in these tissues.

## Supporting Information

Figure S1 **Determination of the optimal time for carrying out the microarray.** Adipocytes differentiated from the fibroblast cell line 3T3-L1 were incubated with 50 *C. burnetii* per cell for 2, 4, 8, and 24 hours and the expression of *TNF* (white bars), *IL6* (black bars), and *CCL2* (gray bars) genes was quantified using qRT-PCR. Results are expressed as the ration of expression levels in stimulated adipocytes *vs* unstimulated adipocytes (mean ±SD, n = 6 per time point).
(TIF)

Figure S2 **Transcriptional profile in adipocytes after stimulation with heat-killed** ***C. burnetii.*** Adipocytes differentiated from the fibroblast cell line 3T3-L1 were incubated with 50 heat-killed *C. burnetii* (HK C. b) per cell for 8 hours and the expression of a set of genes was quantified using qRT-PCR. Results are expressed as the ration of expression levels in HK C. b-stimulated adipocytes *vs* unstimulated adipocytes (mean ±SD, n = 6 per time point).
(TIF)

Figure S3 **Gene expression in infected AT.** Abdominal, inguinal and dorsal AT from BALB/c mice inoculated via the IP route were sampled at day 4, 8, 15 and 30 p.i. The expression of several genes was quantified by qRT-PCR. Results are expressed as the ration of expression levels in infected tissues *vs* tissues from uninfected mice (mean ±SD, n = 3 per time point).
(TIF)

Table S1 **Sequences of specific primers used for qRT-PCR.**
(DOCX)

Table S2 **The complete list of modulated probes.** The response of cultured adipocytes to *C. burnetii* stimulation was studied using microarray analysis: 600 probes (466 genes) were significantly modulated (FC≥1.5 and FDR<0.05). Green color: 468 probes down-modulated. Orange color: 132 probes up-modulated.
(DOCX)
