# Supplementary figures and images for: Persistence of Coxiella burnetii, the Agent of Q Fever, in Murine Adipose Tissue
Source: PLoS One. 2014 May 16;9(5):e97503. doi: 10.1371/journal.pone.0097503 (PMC4023977; doi:10.1371/journal.pone.0097503)

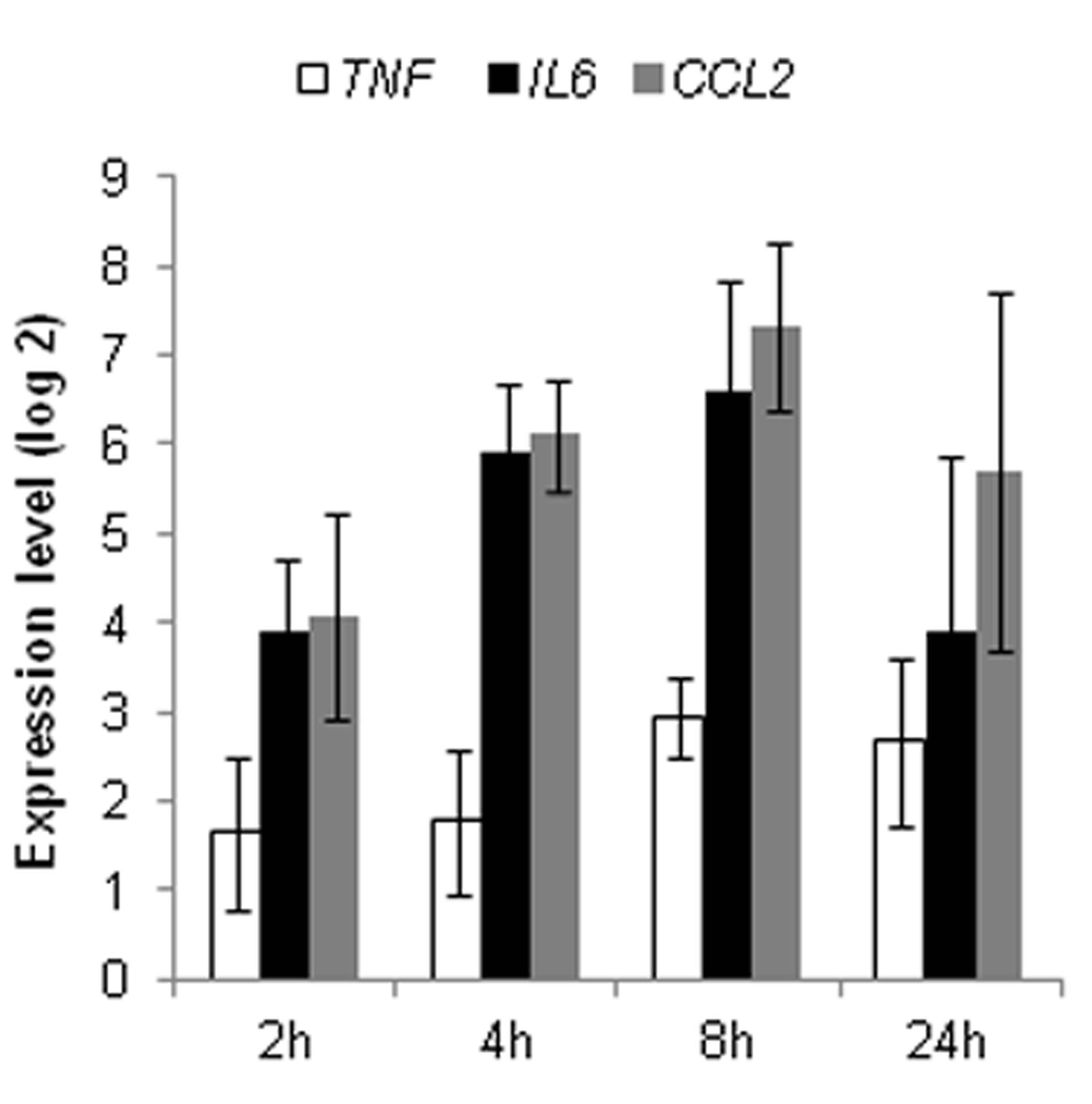

Supplement: Figure S1 — Determination of the optimal time for carrying out the microarray. Adipocytes differentiated from the fibroblast cell line 3T3-L1 were incubated with 50 C. burnetii per cell for 2, 4, 8, and 24 hours and the expression of TNF (white bars), IL6 (black bars), and CCL2 (gray bars) genes was quantified using qRT-PCR. Results are expressed as the ration of expression levels in stimulated adipocytes vs unstimulated adipocytes (mean ±SD, n = 6 per time point). (TIF) [file pone.0097503.s001.tif]

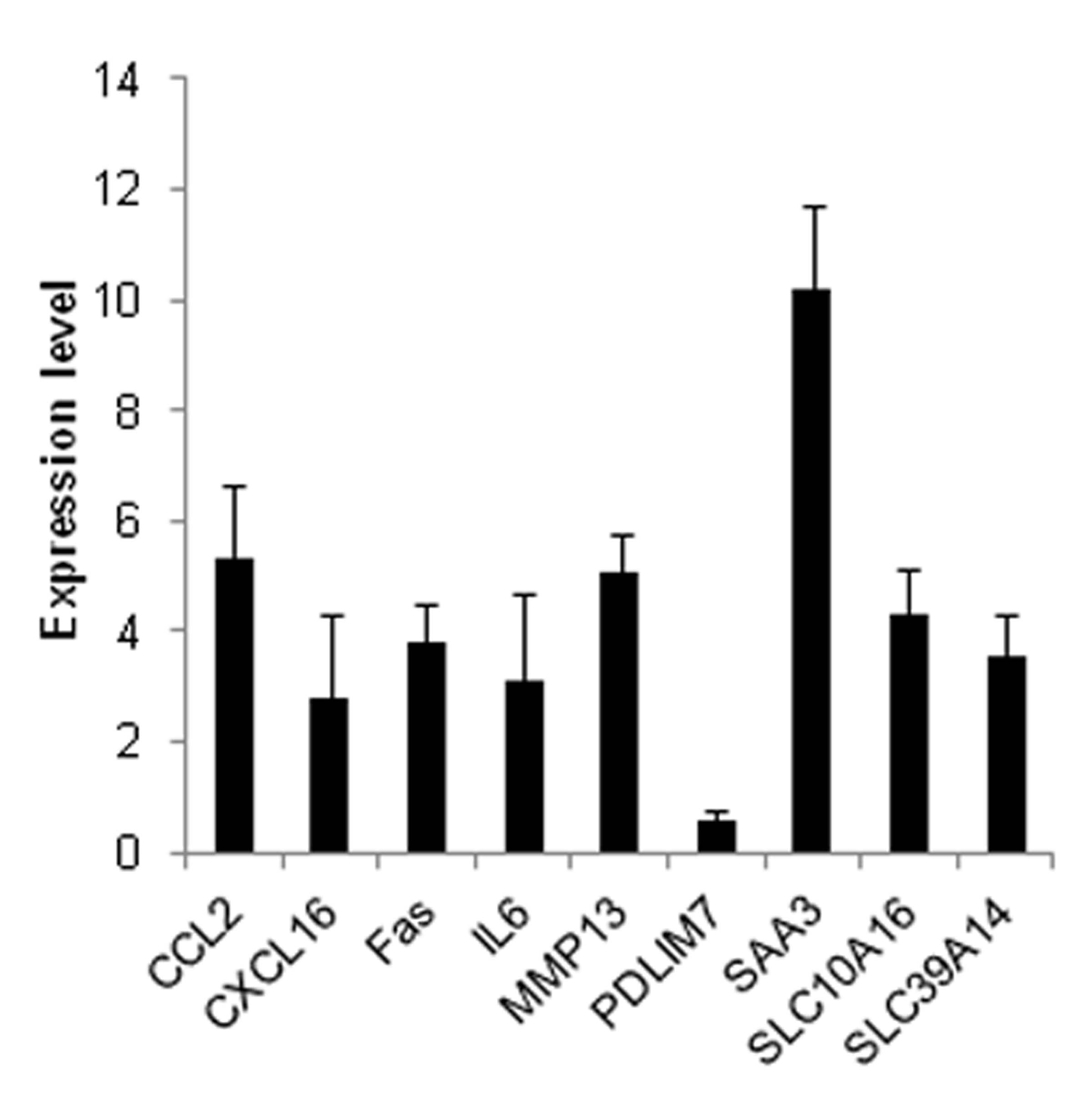

Supplement: Figure S2 — Transcriptional profile in adipocytes after stimulation with heat-killed C. burnetii. Adipocytes differentiated from the fibroblast cell line 3T3-L1 were incubated with 50 heat-killed C. burnetii (HK C. b) per cell for 8 hours and the expression of a set of genes was quantified using qRT-PCR. Results are expressed as the ration of expression levels in HK C. b-stimulated adipocytes vs unstimulated adipocytes (mean ±SD, n = 6 per time point). (TIF) [file pone.0097503.s002.tif]

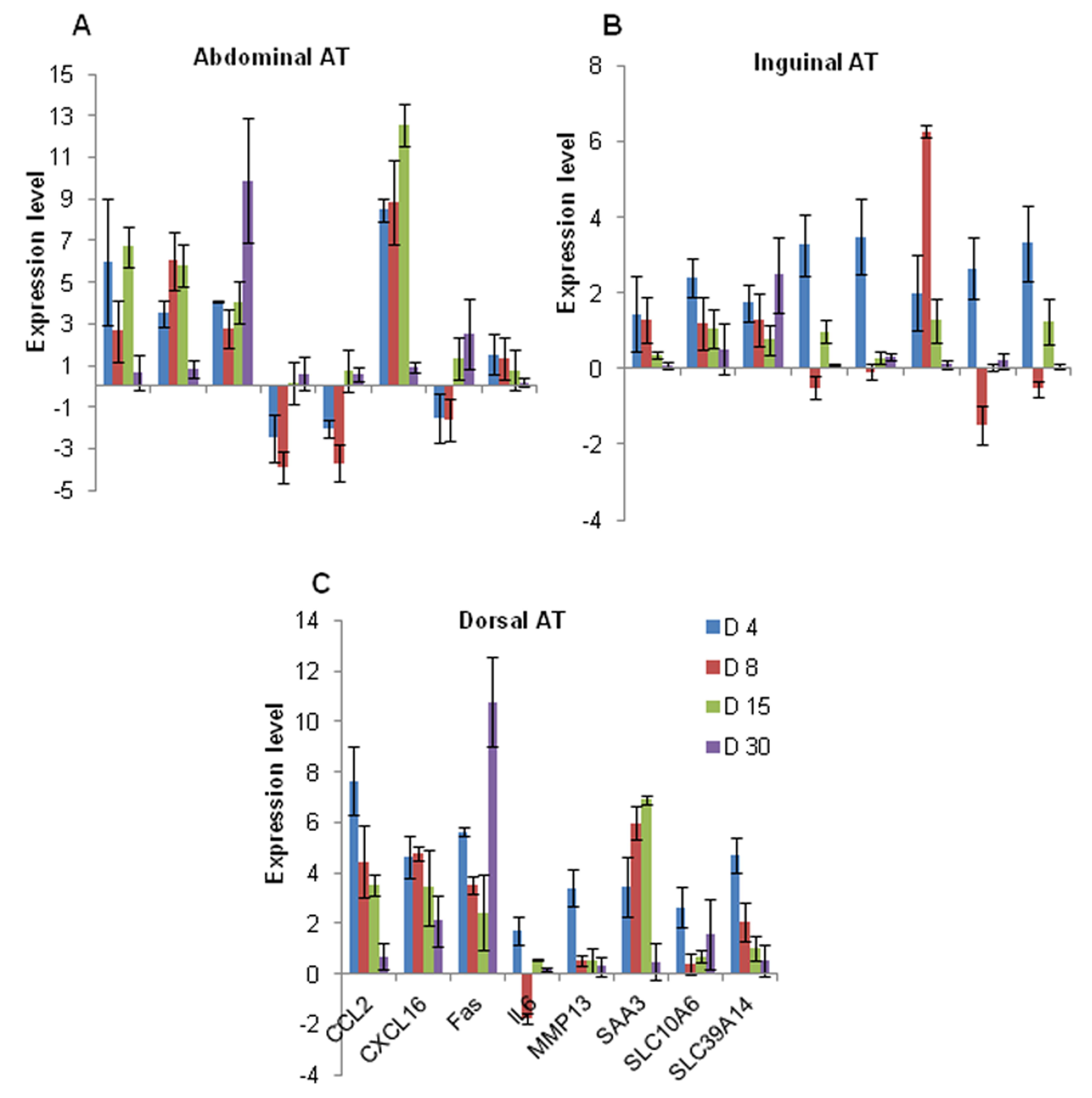

Supplement: Figure S3 — Gene expression in infected AT. Abdominal, inguinal and dorsal AT from BALB/c mice inoculated via the IP route were sampled at day 4, 8, 15 and 30 p.i. The expression of several genes was quantified by qRT-PCR. Results are expressed as the ration of expression levels in infected tissues vs tissues from uninfected mice (mean ±SD, n = 3 per time point). (TIF) [file pone.0097503.s003.tif]
