# Supplementary material for: Persistence of Coxiella burnetii, the Agent of Q Fever, in Murine Adipose Tissue
Source: PLoS One. 2014 May 16;9(5):e97503. doi: 10.1371/journal.pone.0097503 (PMC4023977; doi:10.1371/journal.pone.0097503)
Supplement: Table S1 — Sequences of specific primers used for qRT-PCR. (DOCX) [file pone.0097503.s004.docx]

**Table S1**. Sequences of specific primers used for qRT-PCR

| Gene symbol | 5' primer (5'-3') | 3' primer (5'-3') |
| --- | --- | --- |
| β−actin | tggaatcctgtggcatccatgaaaca | taaaacgcagctcagtaacagtccg |
| TNF | aacagaaacacaagatgctgg | tctggaaaggtctgaaggtagga |
| CCL2 | gaggtggttgtggaaaaggtag | cccaatgagtaggctggagag |
| IL6 | ctctgaaggactctggctttgt | gggaaatcgtggaaatgaga |
| CXCL16 | atgaccagttccacactctttg | gtctcttgcgttcttccttttc |
| Fas | agtgtctggggttgattttcc | caaggagggcaagatagatgag |
| SLC39A14 | ctgtcgttcttctcatcctcct | gcaggctctcttcttcaacttc |
| SLC10A6 | tgatacggaatgacgaggttct | gggcaccatctctaatgttctc |
| SAA3 | actgggaacaacaggaagagaa | atgctcgggggaactatga |
| MMP13 | ggcatttccctttatctgtgc | acctgattcttgcgtgctatg |
| PDLIM7 | tattgcgagcgagactatgaga | gcctgtccttcttggagtagaa |
