# Supplementary material for: Persistence of Coxiella burnetii, the Agent of Q Fever, in Murine Adipose Tissue
Source: PLoS One. 2014 May 16;9(5):e97503. doi: 10.1371/journal.pone.0097503 (PMC4023977; doi:10.1371/journal.pone.0097503)
Supplement: Table S2 — The complete list of modulated probes. The response of cultured adipocytes to C. burnetii stimulation was studied using microarray analysis: 600 probes (466 genes) were significantly modulated (FC≥1.5 and FDR<0.05). Green color: 468 probes down-modulated. Orange color: 132 probes up-modulated. (DOCX) [file pone.0097503.s005.docx]

**Table S2**. The complete list of modulated probes

| **PROBE_ID** | **SYMBOL** | **FC** | **ALIASES** |
| --- | --- | --- | --- |
| A_52_P337086 | Thbs1 | 0.2 | TSP-1. TSP1. Thbs-1. Thbs1. tbsp1 |
| A_52_P337092 | Thbs1 | 0.21 | TSP-1. TSP1. Thbs-1. Thbs1. tbsp1 |
| A_51_P283456 | Cyp2e1 | 0.26 | Cyp2e. Cyp2e1 |
| A_52_P210078 | Acta2 | 0.27 | 0610041G09Rik. Acta2. Actvs. SMalphaA. a-SMA. alphaSMA |
| A_51_P140237 | Fhl2 | 0.28 | C76204. Fhl2. SLIM3 |
| A_52_P363951 | Thbs1 | 0.3 | TSP-1. TSP1. Thbs-1. Thbs1. tbsp1 |
| A_52_P517683 | Tagln | 0.32 | Sm22. Sm22a. Tagln. Ws310 |
| A_51_P463765 | Timp3 | 0.32 | Timp-3. Timp3 |
| A_51_P157042 | Ctgf | 0.33 | Ccn2. Ctgf. Fisp12. Hcs24. fisp-12 |
| A_51_P514070 | Megf10 | 0.33 | 3000002B06Rik. Gm331. Megf10 |
| A_51_P308298 | Myl9 | 0.35 | AI327049. MLC20. Myl9. Mylc2c. RLC-C |
| A_52_P65496 | Iqgap2 | 0.35 | 4933417J23Rik. A630053O10. AI788777. Iqgap2 |
| A_51_P269404 | Fmo3 | 0.36 | AW111792. Fmo3 |
| A_51_P327751 | Ifit1 | 0.36 | ISG56. Ifi56. Ifit1 |
| A_52_P65494 | Iqgap2 | 0.37 | 4933417J23Rik. A630053O10. AI788777. Iqgap2 |
| A_52_P157726 | Ear7 | 0.38 | Ear7. mR7 |
| A_52_P11817 | Fn1 | 0.39 | E330027I09. Fn. Fn-1. Fn1. MGC117493 |
| A_51_P268535 | Ear10 | 0.4 | Ear10 |
| A_51_P273639 | Slc7a5 | 0.4 | 4F2LC. D0H16S474E. Slc7a5. TA1 |
| A_52_P565507 | Ear3 | 0.4 | Ear3. Rnase3. mR3 |
| A_51_P379293 | Ear6 | 0.4 | Ear6 |
| A_51_P272106 | Cirbp | 0.41 | Cirbp. Cirp. R74941 |
| A_51_P207706 | Fam180a | 0.41 | B230314O19. Fam180a |
| A_51_P492339 | Cyp2b13 | 0.41 | Cyp2b13 |
| A_51_P301930 | Lrrc17 | 0.41 | 37kDa. 4833425M04Rik. 6130400C22Rik. Lrrc17. P37nb |
| A_51_P467076 | Cyp2b9 | 0.41 | Cyp2b. Cyp2b9 |
| A_52_P367520 | Nexn | 0.41 | 1110046H09Rik. AA553326. NELIN. Nexn |
| A_51_P326932 | Prune2 | 0.41 | 6330414G02Rik. A230083H22Rik. A330102H22Rik. Bmcc1. KIAA0367. Prune2. mKIAA0367 |
| A_52_P326548 | Fhod3 | 0.41 | A930009H06Rik. FHOS2. Fhod3. KIAA1695. mKIAA1695 |
| A_51_P335000 | Fhl1 | 0.42 | Fhl1. KyoT |
| A_52_P421713 | 9930012K11Rik | 0.42 | 9930012K11Rik. AU016692. MGC91159 |
| A_51_P139985 | Rgr | 0.42 | Rgr |
| A_52_P420504 | Acta2 | 0.43 | 0610041G09Rik. Acta2. Actvs. SMalphaA. a-SMA. alphaSMA |
| A_52_P282058 | Col8a1 | 0.43 | Col8a-1. Col8a1 |
| A_52_P412585 | Ear1 | 0.44 | EAR-1. ECP 1. Ear1. MGC151437 |
| A_52_P285024 | Sertad4 | 0.44 | 4833404A09. C130018M11Rik. Sertad4 |
| A_52_P87900 | Fam107a | 0.44 | DRR1. Fam107a. MGC58343 |
| A_52_P5891 | NA | 0.44 |  |
| A_51_P100856 | Fn1 | 0.45 | E330027I09. Fn. Fn-1. Fn1. MGC117493 |
| A_51_P144264 | Klf2 | 0.45 | Klf2. Lklf |
| A_51_P355629 | Gas2 | 0.45 | Gas-2. Gas2 |
| A_52_P34979 | Epc1 | 0.45 | 2400007E14Rik. 5730566F07Rik. A930032N02Rik. Epc1 |
| A_51_P144801 | 2210403K04Rik | 0.45 | 2210403K04Rik |
| A_51_P449777 | Pmepa1 | 0.46 | 2210418I02Rik. AW455466. Erg1.2. N4wbp4. Pmepa1. Stag1. Tmepai |
| A_51_P134812 | Chac1 | 0.46 | 1810008K03Rik. Chac1 |
| A_51_P115005 | Edn1 | 0.46 | ET-1. Edn1. preproET |
| A_51_P427663 | Cnn2 | 0.46 | AA408047. AI324678. Calpo2. Cnn2 |
| A_52_P5454 | Cd248 | 0.46 | 2610111G01Rik. AI842296. Cd164l1. Cd248. Tem1 |
| A_52_P75034 | 9930012K11Rik | 0.46 | 9930012K11Rik. AU016692. MGC91159 |
| A_52_P675530 | Fat4 | 0.46 | 6030410K14Rik. 9430004M15. Fat4 |
| A_51_P353056 | Fat4 | 0.46 | 6030410K14Rik. 9430004M15. Fat4 |
| A_52_P433011 | NA | 0.47 |  |
| A_52_P288251 | Tmem204 | 0.47 | Tmem204 |
| A_52_P472486 | Cyp2b10 | 0.47 | Cyp2b. Cyp2b10. Cyp2b20. p16 |
| A_52_P670026 | Rsad2 | 0.47 | 2510004L01Rik. Rsad2. Vig1. cig5 |
| A_52_P420792 | Txnip | 0.47 | 1200008J08Rik. AA682105. Hyplip1. THIF. Tbp-2. Txnip. VDUP1 |
| A_52_P650855 | Myo1d | 0.48 | 9930104H07Rik. AW544947. D11Ertd9e. MGC183676. Myo1d. myosin-1d |
| A_52_P283910 | Hnrpdl | 0.48 | AA407431. AA959857. D5Ertd650e. D5Wsu145e. Hnrpdl. JKTBP. hnRNP DL. hnRNP-DL |
| A_51_P149469 | Actc1 | 0.48 | Actc-1. Actc1 |
| A_51_P488910 | 4831426I19Rik | 0.48 | 4831426I19Rik. MGC106312. nesprin-3 |
| A_51_P309920 | Itga8 | 0.48 | AI447669. Itga8 |
| A_52_P601446 | Trim27 | 0.48 | AW538890. Gm19403. Rfp. Trim27 |
| A_52_P662873 | Cyth3 | 0.48 | AI648983. ARNO3. CLM3. Cyth3. Grp1. KIAA4241. Pscd3 |
| A_52_P491544 | Nedd9 | 0.49 | Cas-L. CasL. E230025G09Rik. HEF1. Nedd9 |
| A_51_P142621 | Msrb3 | 0.49 | D430026P16Rik. I-8-23. MsrB2. Msrb3 |
| A_51_P342926 | Omd | 0.49 | OSAD. Omd. SLRR2C |
| A_51_P159352 | Sub1 | 0.49 | AI842364. P15. P9. Pc4. Rpo2tc1. Sub1 |
| A_51_P297131 | Vcl | 0.49 | 9430097D22. AA571387. AI462105. AW545629. Vcl |
| A_51_P450527 | Tagln | 0.49 | Sm22. Sm22a. Tagln. Ws310 |
| A_52_P120037 | Emp1 | 0.5 | Emp1. TMP |
| A_51_P262773 | Cdh2 | 0.5 | CDHN. Cdh2. N-cadherin. Ncad |
| A_52_P209383 | Tgfb1i1 | 0.5 | ARA55. Hic5. TSC-5. Tgfb1i1. hic-5 |
| A_51_P351896 | Fam198b | 0.5 | 1110032E23Rik. 2210419I08Rik. AV011458. Ened. Fam198b |
| A_51_P383689 | Fkbp5 | 0.5 | 51kDa. D17Ertd592e. Dit1. FKBP-5. FKBP51. Fkbp5 |
| A_52_P225370 | 2610201A13Rik | 0.5 | 2610201A13Rik. AW488471 |
| A_52_P403443 | NA | 0.5 |  |
| A_52_P119947 | Fam20a | 0.5 | AI606893. Fam20a |
| A_52_P145993 | NA | 0.5 |  |
| A_52_P625321 | Cabp4 | 0.51 | 2410038D05Rik. Cabp4 |
| A_52_P558259 | Dtna | 0.51 | 2210407P21Rik. A0. Dtn. Dtna. a-DB-1. adbn |
| A_51_P366344 | Tgfb1i1 | 0.51 | ARA55. Hic5. TSC-5. Tgfb1i1. hic-5 |
| A_51_P513803 | Fbxo4 | 0.51 | 1700096C12Rik. AI851261. AW494535. Fbx4. Fbxo4 |
| A_52_P413756 | Pi16 | 0.51 | 1200009H11Rik. Cripi. PI-16. Pi16 |
| A_51_P380309 | Ncam1 | 0.51 | CD56. E-NCAM. NCAM-1. Ncam. Ncam1 |
| A_51_P123655 | Kera | 0.51 | CNA2. Kera. SLRR2B |
| A_51_P313711 | Myh9 | 0.51 | C80049. D0Jmb2. E030044M24Rik. Fltn. Myh9. Myhn-1. Myhn1. NMHC II-A. NMHCIIA. NMMHC II-a. NMMHC-A. NMMHC-IIA. TU72.6 |
| A_52_P555789 | Pcdh18 | 0.51 | 3110038E07Rik. BB095589. PCDH68L. Pcdh18 |
| A_51_P461138 | Pdlim1 | 0.51 | Clim1. Pdlim1. mClim1 |
| A_52_P331043 | C430049B03Rik | 0.51 | 2700063P19Rik. 9430052C07Rik. C430049B03Rik |
| A_52_P269158 | Pid1 | 0.52 | 5033414K04Rik. MGC90850. NYGGF4. Pid1 |
| A_51_P314701 | Cyth1 | 0.52 | CLM1. CTH-1. CYTIP. Cyth1. KIAA4240. Pscd1. mKIAA4240 |
| A_52_P29583 | Hipk2 | 0.52 | 1110014O20Rik. B230339E18Rik. Hipk2. Stank |
| A_51_P134475 | Pmepa1 | 0.52 | 2210418I02Rik. AW455466. Erg1.2. N4wbp4. Pmepa1. Stag1. Tmepai |
| A_52_P305685 | NA | 0.52 |  |
| A_51_P235816 | Tsc22d3 | 0.52 | DIP. Dsip1. Gilz. TSC-22R. Tilz3. Tsc22d3 |
| A_52_P418884 | Nedd9 | 0.52 | Cas-L. CasL. E230025G09Rik. HEF1. Nedd9 |
| A_51_P124535 | Mest | 0.52 | AA408879. AI256745. Mest. Peg1 |
| A_51_P309234 | NA | 0.52 |  |
| A_51_P265806 | Clca2 | 0.52 | Clca2 |
| A_51_P430929 | Fam20a | 0.52 | AI606893. Fam20a |
| A_52_P382325 | Ahnak | 0.52 | 1110004P15Rik. 2310047C17Rik. AA589382. AV091586. Ahnak. DY6 |
| A_52_P604149 | Adamts6 | 0.52 | 5031426K13. A930019D11Rik. ADAM-TS6. Adamts6 |
| A_51_P455338 | Ear11 | 0.52 | Ear11 |
| A_52_P55772 | Tbxa2r | 0.53 | MGC107665. TP. TXA2. Tbxa2r |
| A_51_P428082 | Spnb2 | 0.53 | 9930031C03Rik. AL033301. KIAA4049. SPTB2. SPTBN1. Spnb-2. Spnb2. elf1. elf3. mKIAA4049 |
| A_51_P459465 | Kidins220 | 0.53 | 3110039L19Rik. AI194387. AI316525. C330002I19Rik. Kidins220. mKIAA1250 |
| A_52_P375970 | Fam38b | 0.53 | 5930434P17. 9030411M15Rik. 9430028L06Rik. FLJ23403. Fam38b. Fam38b2. MGC182702. Piezo2 |
| A_51_P253732 | NA | 0.53 |  |
| A_52_P306065 | Nuak1 | 0.53 | AU014801. AW494241. B230104P22Rik. MGC90816. Nuak1. Omphk1 |
| A_52_P576230 | Tacc2 | 0.53 | KIAA4180. Tacc2. mKIAA4180 |
| A_52_P471282 | Fmo4 | 0.53 | D1Ertd532e. Fmo4. MGC124273. MGC124274 |
| A_51_P257675 | Tspyl4 | 0.53 | 2610102M01Rik. B230210I21Rik. D10Bwg0791e. Tspyl4 |
| A_51_P488928 | Cyth3 | 0.53 | AI648983. ARNO3. CLM3. Cyth3. Grp1. KIAA4241. Pscd3 |
| A_51_P167273 | Rpp14 | 0.53 | 2610511E03Rik. AA682089. Rpp14 |
| A_52_P949440 | Tgfb1i1 | 0.54 | ARA55. Hic5. TSC-5. Tgfb1i1. hic-5 |
| A_51_P406583 | Myo1e | 0.54 | 2310020N23Rik. 9130023P14Rik. AA407778. Myo1e. myosin-1e. myr 3 |
| A_51_P405912 | Lmcd1 | 0.54 | AW455500. AW553074. Lmcd1. MGC29184. dyxin |
| A_51_P188271 | Cd248 | 0.54 | 2610111G01Rik. AI842296. Cd164l1. Cd248. Tem1 |
| A_52_P301821 | D0H4S114 | 0.54 | AI325076. D0H4S114. Harp. P311. PTZ17. SEZ17 |
| A_52_P160328 | Angpt1 | 0.54 | 1110046O21Rik. Ang-1. Ang1. Angpt1 |
| A_52_P121491 | Msrb3 | 0.54 | D430026P16Rik. I-8-23. MsrB2. Msrb3 |
| A_52_P615952 | Qk | 0.54 | 1110003F05Rik. 1500005P18. Qk. QkI. l(17)-1Wis. l17Wis1 |
| A_51_P472726 | Pdlim2 | 0.54 | 4732462F18Rik. MGC37634. Pdlim2. Slim |
| A_51_P264769 | Dmp1 | 0.54 | AV020965. Dmp. Dmp1. MGC130441. PP |
| A_51_P472829 | Aif1l | 0.55 | 2810003C17Rik. AI043124. Aif1l. C87647. Iba2. MGC30545 |
| A_51_P499599 | Osr2 | 0.55 | 5430409I15Rik. Osr2. Osr2A. Osr2B |
| A_52_P257919 | NA | 0.55 |  |
| A_51_P514085 | Mx2 | 0.55 | AI528743. Mx-2. Mx2 |
| A_52_P109740 | NA | 0.55 |  |
| A_52_P259537 | Gck | 0.55 | GLK. Gck. Gk. HK4. HKIV. HXKP. MODY2 |
| A_52_P247086 | Rprd2 | 0.55 | 2810036A19Rik. 4930535B03Rik. 6720469I21Rik. AL022841. AL022940. AU021304. BB077382. Rprd2. mKIAA0460 |
| A_52_P6045 | Gpsm2 | 0.55 | 6230410J09Rik. Gpsm2. LGN. Pins |
| A_51_P449706 | Lpp | 0.55 | 9430020K16Rik. AA959454. AU024130. B130055L10Rik. C79715. D630048H16. Lpp |
| A_52_P249672 | 4833417J20Rik | 0.55 | 4833417J20Rik |
| A_52_P136162 | Fgfr1 | 0.55 | AW208770. FLG. Fgfr-1. Fgfr1. Flt-2. Hspy |
| A_52_P44949 | Tpm1 | 0.55 | AA986836. AI854628. TM2. Tm3. Tmpa. Tpm-1. Tpm1. alpha-TM |
| A_51_P221512 | Ppp1r2 | 0.55 | 0610025N14Rik. 2310007G06Rik. 4930440J04Rik. 5430408E15Rik. D16Ertd248e. IPP-2. Ppp1r2 |
| A_52_P325477 | Trim16 | 0.55 | 9130006M08Rik. AI482483. EBBP. Trim16 |
| A_51_P356760 | Mical1 | 0.55 | MGC27831. MGC38321. MICAL. Mical1. Nical |
| A_51_P235821 | Tsc22d3 | 0.55 | DIP. Dsip1. Gilz. TSC-22R. Tilz3. Tsc22d3 |
| A_51_P301508 | Ski | 0.55 | 2310012I02Rik. 2610001A11Rik. AA062172. AA589460. BC004088. MGC8300. Ski |
| A_51_P303089 | Ttc28 | 0.55 | 2310015L07Rik. 6030435N04. AI428795. AI851761. BC002262. MGC7623. Ttc28 |
| A_52_P532910 | Tpm1 | 0.55 | AA986836. AI854628. TM2. Tm3. Tmpa. Tpm-1. Tpm1. alpha-TM |
| A_52_P312904 | Pcbp2 | 0.55 | AW412548. Hnrpx. MGC107004. Pcbp2. alphaCP-2 |
| A_51_P384469 | Syncrip | 0.55 | 2610109K23Rik. 4632417O19Rik. GRY-RBP. Nsap1. Nsap1l. Syncrip. pp68 |
| A_52_P174942 | Nrp1 | 0.55 | C530029I03. NP-1. NPN-1. Npn1. Nrp. Nrp1 |
| A_52_P63709 | Fam49b | 0.55 | 0910001A06Rik. AW122079. Fam49b |
| A_51_P451052 | Pgm5 | 0.55 | 4833423B07. 9530034F03Rik. D830025G17. Pgm5. aciculin |
| A_51_P143468 | Klhl26 | 0.55 | C630013N10Rik. Klhl26. Klkl26. MGC38024 |
| A_52_P164821 | NA | 0.55 |  |
| A_51_P486639 | NA | 0.55 |  |
| A_52_P365660 | Lrrc4c | 0.56 | 6430556C10Rik. Lrrc4c. NGL-1. mKIAA1580 |
| A_51_P450957 | Acta2 | 0.56 | 0610041G09Rik. Acta2. Actvs. SMalphaA. a-SMA. alphaSMA |
| A_51_P374571 | Igfbp6 | 0.56 | IGFBP-6. Igfbp6 |
| A_52_P157402 | Pdp1 | 0.56 | Gm1024. Pdp1. Ppm2c |
| A_51_P520760 | NA | 0.56 |  |
| A_51_P359570 | Ifit3 | 0.56 | Ifi49. Ifit3. MGC107331 |
| A_51_P189361 | Osgin1 | 0.56 | 1700012B18Rik. Okl38. Osgin1 |
| A_52_P42069 | Prokr1 | 0.56 | EG-VEGFR1. Gpr73. Pkr1. Prokr1 |
| A_52_P190973 | Vcl | 0.56 | 9430097D22. AA571387. AI462105. AW545629. Vcl |
| A_52_P149438 | Pdlim5 | 0.56 | 1110001A05Rik. AI987914. C87059. Enh. Enh1. Enh2. Enh3. LIM. Pdlim5 |
| A_51_P177392 | Tacc2 | 0.56 | KIAA4180. Tacc2. mKIAA4180 |
| A_52_P219753 | Cpt1a | 0.56 | C730027G07. CPTI. Cpt1. Cpt1a |
| A_52_P98140 | NA | 0.56 |  |
| A_52_P473813 | NA | 0.56 |  |
| A_52_P84384 | Flna | 0.56 | ABP-280. Dilp2. F730004A14Rik. Fln1. Flna. filamin-1 |
| A_52_P344354 | Rprd2 | 0.56 | 2810036A19Rik. 4930535B03Rik. 6720469I21Rik. AL022841. AL022940. AU021304. BB077382. Rprd2. mKIAA0460 |
| A_51_P150175 | Ube4a | 0.57 | 4732444G18Rik. 9930123J21Rik. UFD2b. Ube4a |
| A_52_P335064 | Mustn1 | 0.57 | 1110028G01Rik. MGC151316. MGC151318. Mustang. Mustn1 |
| A_51_P205907 | Flnc | 0.57 | 1110055E19Rik. ABP-280. ABPL. Fln2. Flnc |
| A_51_P170807 | Map3k6 | 0.57 | Ask2. MAPKKK6. MEKK6. MGC159280. Map3k6 |
| A_52_P468451 | NA | 0.57 |  |
| A_51_P309917 | Itga8 | 0.57 | AI447669. Itga8 |
| A_52_P665143 | Ccdc45 | 0.57 | 4732496G21Rik. AI118346. AI448335. Ccdc45. Cep95. D330027A14Rik. F630025I20Rik |
| A_52_P441036 | Col23a1 | 0.57 | 2810458L13Rik. AI429655. Col23a1 |
| A_52_P58066 | NA | 0.57 |  |
| A_51_P506128 | Zfp28 | 0.57 | 2810438M17Rik. Zfp-28. Zfp28. mkr-5 |
| A_51_P284177 | Akr1c14 | 0.57 | 9030611N15Rik. AW557061. Akr1c14 |
| A_51_P380778 | A730056A06Rik | 0.57 | A730056A06Rik. C76391 |
| A_51_P315785 | Tnfaip6 | 0.57 | TSG-6. Tnfaip6. Tnfip6. Tsg6 |
| A_52_P647488 | NA | 0.57 |  |
| A_52_P57622 | Acss3 | 0.57 | 8430416H19Rik. Acss3. Gm874 |
| A_52_P156158 | Grpel1 | 0.57 | AA408748. Grpel1. MGC8152. mt-GrpE#1. mt-Grpel1 |
| A_52_P229278 | Limch1 | 0.57 | 3732412D22Rik. Limch1. mKIAA1102 |
| A_51_P342567 | Akap12 | 0.57 | AI317366. Akap12. SSeCKS. Srcs5. Tsga12 |
| A_52_P853177 | Angptl2 | 0.57 | AI593246. AW260363. Angptl2. Arp2 |
| A_52_P418477 | Tpm2 | 0.57 | Tpm-2. Tpm2. Trop-2 |
| A_52_P154710 | Sesn2 | 0.57 | HI95. MGC11758. SEST2. Ses2. Sesn2 |
| A_51_P241269 | Actg2 | 0.57 | ACTA3. Act-4. Act4. Actg2. SMGA |
| A_52_P178151 | Slc6a6 | 0.57 | AA589629. C80501. Slc6a6. Taut |
| A_52_P21574 | Pdlim7 | 0.57 | 1110003B01Rik. 2410002J21Rik. AV007930. Enigma. Pdlim7 |
| A_52_P14666 | NA | 0.57 |  |
| A_51_P267494 | Cdc42ep3 | 0.57 | 3200001F04Rik. AA986861. Borg2. Cdc42ep3. Cep3. UB1 |
| A_51_P432892 | NA | 0.58 |  |
| A_51_P191865 | Lama2 | 0.58 | 5830440B04. KIAA4087. Lama2. dy. mKIAA4087. mer. merosin |
| A_51_P452637 | Pdp1 | 0.58 | Gm1024. Pdp1. Ppm2c |
| A_51_P437240 | Emp2 | 0.58 | Emp2. XMP |
| A_51_P183515 | 2210416O15Rik | 0.58 | 2210416O15Rik |
| A_51_P283473 | Fibin | 0.58 | 1110018M03Rik. Fibin |
| A_51_P502872 | 2200002D01Rik | 0.58 | 2010005J02Rik. 2200002D01Rik. H2RSP. IMUP. IMUP-1. IMUP-2 |
| A_52_P4482 | NA | 0.58 |  |
| A_52_P151393 | AI646023 | 0.58 | AI646023. MGC65625. MGC67483 |
| A_51_P108252 | Gpsm2 | 0.58 | 6230410J09Rik. Gpsm2. LGN. Pins |
| A_51_P183571 | Serpine1 | 0.58 | PAI-1. PAI1. Planh1. Serpine1 |
| A_52_P634090 | Jag1 | 0.58 | Htu. Jag1. Ozz. Ser-1 |
| A_52_P473172 | NA | 0.58 |  |
| A_51_P123895 | D0H4S114 | 0.58 | AI325076. D0H4S114. Harp. P311. PTZ17. SEZ17 |
| A_52_P61735 | Flnc | 0.58 | 1110055E19Rik. ABP-280. ABPL. Fln2. Flnc |
| A_52_P440885 | 9630023C09Rik | 0.58 | 9630023C09Rik |
| A_52_P489193 | Pdlim7 | 0.58 | 1110003B01Rik. 2410002J21Rik. AV007930. Enigma. Pdlim7 |
| A_52_P295104 | 2210020M01Rik | 0.58 | 2210020M01Rik |
| A_52_P622244 | 6030419C18Rik | 0.58 | 6030419C18Rik |
| A_52_P653902 | Eny2 | 0.58 | 1810057B09Rik. 6720481I12. DC6. Eny2. Ey2 |
| A_52_P273098 | Slc1a4 | 0.58 | ASCT-1. ASCT1. AW045657. SATT. Slc1a4 |
| A_52_P247022 | Arhgap20 | 0.58 | 6530403F17Rik. A530023E23Rik. Arhgap20. mKIAA1391 |
| A_52_P653054 | NA | 0.59 |  |
| A_51_P481418 | Arhgap21 | 0.59 | 5530401C11Rik. AA416458. ARHGAP10. Arhgap21 |
| A_52_P499297 | Zmym3 | 0.59 | 9030216B10Rik. AW122925. DXS6673E. DXS6673El. MGC118059. Zfp261. Zmym3 |
| A_52_P617638 | Nox4 | 0.59 | AI648021. Nox4 |
| A_52_P600518 | Tsc22d3 | 0.59 | DIP. Dsip1. Gilz. TSC-22R. Tilz3. Tsc22d3 |
| A_52_P63728 | Osr1 | 0.59 | Odd1. Osr. Osr1 |
| A_52_P432969 | NA | 0.59 |  |
| A_52_P141417 | Dact1 | 0.59 | 4921528D17Rik. AI115603. DAPPER. DAPPER1. Dact1. FRODO. Frd1. Frodo1. MDpr1. MTNG3. THYEX3 |
| A_52_P306537 | C230096C10Rik | 0.59 | 2700016F22Rik. C230096C10Rik. Kiaa0090. mKIAA0090 |
| A_51_P224534 | Ahnak | 0.59 | 1110004P15Rik. 2310047C17Rik. AA589382. AV091586. Ahnak. DY6 |
| A_52_P474719 | Lrrc16a | 0.59 | 1110037D04Rik. AI425970. CARMIL. D130057M20. Lrrc16. Lrrc16a |
| A_52_P600274 | Trib3 | 0.59 | Ifld2. Nipk. SINK. SKIP3. TRB-3. Trb3. Trib3 |
| A_52_P613255 | Ncam1 | 0.59 | CD56. E-NCAM. NCAM-1. Ncam. Ncam1 |
| A_52_P235064 | Ubr5 | 0.59 | 4432411E13Rik. AW549941. C77315. D030042K14. Edd. Edd1. Ubr5. mKIAA0896 |
| A_51_P186703 | Fbln5 | 0.59 | A55. DANCE. EVEC. Fbln5 |
| A_52_P195839 | Ctsc | 0.59 | AI047818. Ctsc. DPP1. DPPI |
| A_51_P462837 | NA | 0.59 |  |
| A_52_P248343 | Mylip | 0.59 | 9430057C20Rik. MGC11702. Mir. Mylip |
| A_51_P262437 | Srsf2 | 0.59 | D11Wsu175e. MRF-1. Pr264. SC35. Sfrs10. Sfrs2. Srsf2 |
| A_52_P217492 | 5031425E22Rik | 0.59 | 5031425E22Rik |
| A_52_P531325 | Rps6 | 0.59 | MGC102571. MGC103209. Rps6 |
| A_51_P115441 | Dpysl3 | 0.59 | CRMP-4. Dpysl3. TUC4. Ulip. Ulip1 |
| A_51_P471520 | Stk25 | 0.59 | 1500019J11Rik. AU018434. C86992. Ste20-like. Stk25. Ysk1 |
| A_52_P373782 | Plaa | 0.59 | 2410007N06. AI536418. AU018445. AW208417. D4Ertd618e. PLA2P. PLAP. Plaa. Ufd3 |
| A_51_P363681 | Mfap5 | 0.59 | MAGP-2. Mfap5 |
| A_51_P365980 | 2310022B05Rik | 0.59 | 2310022B05Rik |
| A_51_P222337 | Rspo2 | 0.6 | 2610028F08Rik. AA673245. D430027K22. Rspo2. ftls |
| A_51_P177667 | Fyn | 0.6 | AI448320. AW552119. Fyn. MGC115870 |
| A_52_P564663 | NA | 0.6 |  |
| A_51_P494293 | 9130024F11Rik | 0.6 | 9130024F11Rik. B530002L08. EG329160 |
| A_52_P219943 | Epc1 | 0.6 | 2400007E14Rik. 5730566F07Rik. A930032N02Rik. Epc1 |
| A_52_P512955 | Anln | 0.6 | 1110037A17Rik. 2900037I21Rik. Anln. C78101. Scraps |
| A_51_P453818 | C230098O21Rik | 0.6 | AI836570. AW557042. C230098O21Rik |
| A_52_P303841 | Gm5296 | 0.6 | Gm5296 |
| A_51_P442032 | Cask | 0.6 | Cask. DXPri1. DXRib1. LIN-2. MGC7449. Pals3. mLin-2 |
| A_51_P220343 | Wisp1 | 0.6 | AW146261. CCN4. Elm1. Wisp1 |
| A_51_P109469 | Fgfr1 | 0.6 | AW208770. FLG. Fgfr-1. Fgfr1. Flt-2. Hspy |
| A_52_P339996 | Slc6a6 | 0.6 | AA589629. C80501. Slc6a6. Taut |
| A_51_P364168 | Lrp5 | 0.6 | BMND1. HBM. KIAA4142. LR3. LRP7. Lrp5. OPPG. mKIAA4142 |
| A_51_P106294 | Gck | 0.6 | GLK. Gck. Gk. HK4. HKIV. HXKP. MODY2 |
| A_51_P303095 | Ttc28 | 0.6 | 2310015L07Rik. 6030435N04. AI428795. AI851761. BC002262. MGC7623. Ttc28 |
| A_51_P181571 | Hnrnpl | 0.6 | C79783. D830027H13Rik. Hnrnpl. Hnrpl |
| A_52_P63044 | Lsp1 | 0.6 | Lsp-1. Lsp1. WP34. p50. pp52 |
| A_52_P74441 | NA | 0.6 |  |
| A_52_P222775 | Map3k6 | 0.6 | Ask2. MAPKKK6. MEKK6. MGC159280. Map3k6 |
| A_52_P241484 | Rcl1 | 0.6 | 2310040A02Rik. AI789745. C76567. RPCL1. Rcl1. Rnac |
| A_52_P571691 | Fam178a | 0.6 | 3632432H19. 6030443O07Rik. AU014917. Fam178a |
| A_51_P482908 | Arrdc4 | 0.6 | 2410003C09Rik. AV216361. Arrdc4 |
| A_52_P258507 | Ppp1r10 | 0.6 | 2610025H06Rik. Cat53. D17Ertd808e. Fb19. Pnuts. Ppp1r10 |
| A_51_P327920 | St7l | 0.6 | MGC130399. St7l. St7r |
| A_52_P610900 | Cask | 0.6 | Cask. DXPri1. DXRib1. LIN-2. MGC7449. Pals3. mLin-2 |
| A_52_P1084604 | Wac | 0.6 | 1110067P07Rik. A230035H12Rik. AI256735. AI256776. Wac. Wwp4 |
| A_52_P410609 | Rab31 | 0.6 | 1700093E07Rik. AI415285. Rab22B. Rab31 |
| A_51_P256884 | Mtss1l | 0.6 | ABBA. MGC79213. Mtss1l |
| A_51_P251154 | NA | 0.6 |  |
| A_52_P19606 | Osbpl1a | 0.6 | G430090F17Rik. Gm753. Osbpl1a. Osbpl1b |
| A_51_P496448 | Cul2 | 0.6 | 1300003D18Rik. 4932411N15Rik. AI327301. Cul2. KIAA4106. mKIAA4106 |
| A_51_P418116 | Tmem119 | 0.6 | AW208946. BC025600. MGC38046. Tmem119. obif |
| A_51_P150722 | Lyst | 0.6 | D13Sfk13. Lyst. beige. bg |
| A_52_P315976 | Tpm2 | 0.6 | Tpm-2. Tpm2. Trop-2 |
| A_51_P262432 | Srsf2 | 0.61 | D11Wsu175e. MRF-1. Pr264. SC35. Sfrs10. Sfrs2. Srsf2 |
| A_52_P574685 | Med14 | 0.61 | 9930001L01Rik. AU041628. Crsp2. ENSMUSG00000073278. Gm641. Med14. ORF1. Trap170 |
| A_51_P440865 | Fam110b | 0.61 | 1700012H17Rik. 5031405P22. AW049855. Fam110b |
| A_52_P429534 | Elavl1 | 0.61 | 2410055N02Rik. Elavl1. HUR. Hua. W91709 |
| A_51_P138502 | Ttc28 | 0.61 | 2310015L07Rik. 6030435N04. AI428795. AI851761. BC002262. MGC7623. Ttc28 |
| A_52_P186937 | Cmpk2 | 0.61 | 1200004E04Rik. Cmpk2. TDKI. Tyki |
| A_52_P28651 | Pvrl1 | 0.61 | AI835281. AW549174. Cd111. HIgR. HveC. PRR. PRR1. Pvrl1. nectin-1 |
| A_52_P661412 | Adora1 | 0.61 | A1-AR. A1AR. A1R. AA1R. AI848715. Adora1. BB176431. Ri |
| A_52_P411430 | NA | 0.61 |  |
| A_52_P17081 | Acer3 | 0.61 | 1110057L18Rik. 5430429L08Rik. AV015045. Acer3. Phca |
| A_52_P269003 | Neo1 | 0.61 | 2610028H22Rik. AI327052. D930014N22Rik. Igdcc2. Neo1 |
| A_51_P513898 | Epb4.1l4a | 0.61 | Epb4.1l4. Epb4.1l4a. Epb41l4a. NBL4 |
| A_52_P600625 | Rad23b | 0.61 | 0610007D13Rik. AV001138. HR23B. Rad23b. mHR23B. p58 |
| A_51_P107302 | Cav1 | 0.61 | Cav. Cav-1. Cav1 |
| A_52_P288177 | Epha4 | 0.61 | 2900005C20Rik. AI385584. Cek8. Epha4. Hek8. Sek. Sek1. Tyro1. rb |
| A_52_P405193 | Prkrir | 0.61 | 2900052B10Rik. Dap4. MGC31389. Prkrir. Rpkrir |
| A_52_P292882 | Dap3 | 0.61 | 4921514D13Rik. DAP-3. Dap3 |
| A_52_P535561 | Txndc5 | 0.61 | AL022641. ERp46. PC-TRP. Txndc5 |
| A_52_P72237 | Actg1 | 0.61 | AL023024. Actg. Actg1. Actl. E51. MGC117491. MGC28612 |
| A_51_P489452 | Cdo1 | 0.61 | 1300002L19Rik. Cdo. Cdo1. D18Ucla3 |
| A_52_P106739 | Ddx46 | 0.61 | 2200005K02Rik. 8430438J23Rik. AI325430. AI957095. Ddx46. MGC116676. MGC31579. mKIAA0801 |
| A_51_P304170 | Rtp4 | 0.61 | 5830458K16Rik. Ifrg28. Rtp4 |
| A_52_P281702 | Igfbp5 | 0.61 | AI256729. AW208790. IGFBP-5. IGFBP-5P. Igfbp5 |
| A_52_P330694 | Tle1 | 0.61 | C230057C06Rik. Estm14. Grg1. Tle1. Tle4l |
| A_52_P399717 | Ccdc50 | 0.61 | 2610529H08Rik. 5730448P06Rik. AW048328. AW546090. C3orf6. Ccdc50. D16Bwg1543e |
| A_51_P265016 | Ypel1 | 0.61 | 0610009L05Rik. 1700016N17Rik. 1700019O22Rik. 4921520K19Rik. 4930511F14Rik. AV118478. Dgl1. Ppil2. Ypel1. mdgl-1 |
| A_52_P309337 | Metap2 | 0.61 | 4930584B20Rik. A930035J23Rik. AI047573. AL024412. AU014659. Amp2. MGC102452. Metap2. Mnpep. p67. p67eIF2 |
| A_52_P576854 | Osgin1 | 0.61 | 1700012B18Rik. Okl38. Osgin1 |
| A_52_P68714 | NA | 0.61 |  |
| A_51_P190254 | Scrn1 | 0.61 | 2810019K23Rik. 6330535A03Rik. AI852905. KIAA0193. SES1. Scrn1. mKIAA0193 |
| A_51_P381558 | Rasa4 | 0.61 | AA793972. AW112107. BB079060. CAPRI. GAPL. KIAA0538. Rasa4. mKIAA0538 |
| A_52_P778761 | Vgll3 | 0.62 | 1700110N18Rik. 4832416J22. C80713. Vgl-3. Vgll3. Vito-2 |
| A_51_P203182 | Apobr | 0.62 | Apob-48r. Apob48r. Apobr |
| A_51_P476091 | NA | 0.62 |  |
| A_51_P331570 | Trib3 | 0.62 | Ifld2. Nipk. SINK. SKIP3. TRB-3. Trb3. Trib3 |
| A_51_P280906 | Jag1 | 0.62 | Htu. Jag1. Ozz. Ser-1 |
| A_52_P482355 | Srsf2 | 0.62 | D11Wsu175e. MRF-1. Pr264. SC35. Sfrs10. Sfrs2. Srsf2 |
| A_52_P323621 | NA | 0.62 |  |
| A_51_P502580 | Trim16 | 0.62 | 9130006M08Rik. AI482483. EBBP. Trim16 |
| A_52_P53596 | Sesn1 | 0.62 | 1110002G11Rik. AU044290. MGC118148. Pa26. Sesn1. Sest1 |
| A_51_P302358 | Ltb | 0.62 | AI662801. LTbeta. Ltb. Tnfc. Tnfsf3. p33 |
| A_52_P685999 | Mettl7b | 0.62 | 0610006F02Rik. AI266817. Mettl7b |
| A_52_P423128 | Arglu1 | 0.62 | 9430010O03Rik. Arglu1. C130008N12 |
| A_52_P104769 | Fmnl3 | 0.62 | 2700073B04Rik. FBP11. Fmnl3. Wbp3. mKIAA2014 |
| A_52_P8043 | Srsf2 | 0.62 | D11Wsu175e. MRF-1. Pr264. SC35. Sfrs10. Sfrs2. Srsf2 |
| A_51_P369200 | Tpx2 | 0.62 | 2610005B21Rik. DIL2. REPP86. Tpx2. p100 |
| A_52_P630687 | NA | 0.62 |  |
| A_51_P452876 | Ak1 | 0.62 | Ak-1. Ak1. B430205N08Rik |
| A_52_P419298 | Lasp1 | 0.62 | AA408629. Def-4. Lasp1. SH3P6. Tg(Col1a1-lacZ)1Ngma |
| A_52_P683306 | Fam13b | 0.62 | 2610024E20Rik. AW060714. AW546153. Fam13b. MGC37079 |
| A_52_P309737 | Cdk12 | 0.62 | 1810022J16Rik. AI646528. Cdk12. Crk7. Crkrs. D11Ertd752e. MGC141525. Pksc |
| A_52_P241676 | Slc9a3r2 | 0.62 | 0610011L07Rik. 1200011K07Rik. 2010007A20Rik. E3karp. NHERF-2. Octs2. Sip-1. Sip1. Slc9a3r2. Sryip1. Tka-1 |
| A_51_P142813 | Aspn | 0.62 | 4631401G09Rik. AA986886. Aspn. Plap1. Slrr1c |
| A_51_P293982 | Plekho2 | 0.62 | AI840980. MGC30448. Plekho2. Plekhq1 |
| A_52_P229959 | Sept6 | 0.62 | 2810035H17Rik. C920001C06Rik. KIAA0128. Sep6. Sept6. mKIAA0128 |
| A_52_P317230 | Hdgfrp3 | 0.62 | 2700022B06Rik. HRP-3. Hdgfrp3 |
| A_51_P231440 | Fgfbp3 | 0.63 | 2610306H15Rik. Fgf-bp3. Fgfbp3 |
| A_52_P574291 | Chic2 | 0.63 | 1700081B18Rik. 4930502K01Rik. BTL. Chic2 |
| A_52_P121703 | Gm3625 | 0.63 | Gm3625 |
| A_52_P229648 | Pacsin2 | 0.63 | AI197433. Pacsin2 |
| A_51_P393518 | Hmcn1 | 0.63 | EG545370. Gm201. Hmcn1 |
| A_51_P328926 | Tmpo | 0.63 | 5630400D24Rik. AI195756. AI606875. AW214352. AW547477. LAP2. TP. Tmpo |
| A_51_P473252 | Zyx | 0.63 | 9530098H06Rik. R75157. Zyx |
| A_51_P333839 | Pdgfc | 0.63 | 1110064L01Rik. AI647969. PDGF-C. Pdgfc |
| A_52_P858226 | 2810452K05Rik | 0.63 | 2810452K05Rik |
| A_51_P249180 | Klk1b24 | 0.63 | Klk1b24. Klk24. mGk-24 |
| A_52_P204385 | Lin9 | 0.63 | 2700022J23Rik. AA537062. AA545216. Bara. Lin9. TGS. TGS1 |
| A_51_P179504 | NA | 0.63 |  |
| A_52_P629895 | Adh1 | 0.63 | ADH-AA. AI194826. Adh-1. Adh-1-t. Adh-1e. Adh-1t. Adh-3e. Adh1. Adh1-e. Adh1-t. Adh1tl. Adh3-e |
| A_51_P116137 | Lrig1 | 0.63 | D6Bwg0781e. Img. LIG-1. Lrig1 |
| A_52_P128262 | Clk4 | 0.63 | AI987988. C85119. Clk4 |
| A_52_P706621 | NA | 0.63 |  |
| A_52_P299053 | Ptplb | 0.63 | 6330408J20Rik. AI255777. AI481689. HACD2. Ptplb |
| A_52_P12023 | NA | 0.63 |  |
| A_52_P475805 | Col12a1 | 0.63 | AW743884. Col12a1 |
| A_52_P365434 | D5Ertd579e | 0.63 | 9030221A05Rik. A930018H20Rik. D5Ertd579e. Kiaa0232. mKIAA0232 |
| A_51_P445677 | Hhat | 0.63 | 2810432O22Rik. AI462858. Hhat. MGC11697. Skn |
| A_52_P116327 | NA | 0.63 |  |
| A_52_P826923 | NA | 0.63 |  |
| A_51_P231499 | Enpp1 | 0.63 | 4833416E15Rik. AI428932. C76301. CD203c. E-NPP1. Enpp1. Ly-41. M6S1. NPP1. Npps. PC-1. Pca. Pca-1. Pdnp1. ttw. twy |
| A_51_P375698 | Fam13b | 0.63 | 2610024E20Rik. AW060714. AW546153. Fam13b. MGC37079 |
| A_52_P496397 | Pms2 | 0.63 | AW555130. Pms2. Pmsl2 |
| A_51_P231184 | Anpep | 0.63 | AP-M. AP-N. Anpep. Apn. Cd13. P150 |
| A_51_P386585 | Gpsm1 | 0.63 | 1810037C22Rik. AW107933. Ags3. Gpsm1 |
| A_51_P308844 | Nrn1 | 0.63 | 0710008J23Rik. MGC40786. Nrn. Nrn1. cpg15 |
| A_51_P399664 | Cttn | 0.63 | 1110020L01Rik. Cttn. Ems1 |
| A_51_P373777 | Trim35 | 0.63 | 0710005M05Rik. A430106H13Rik. AW046487. HLS5. Mair. NC8. Trim35. mKIAA1098 |
| A_51_P311694 | Ypel5 | 0.63 | 2310076K21Rik. CGI-127. Ypel5 |
| A_52_P121155 | Pten | 0.63 | 2310035O07Rik. A130070J02Rik. AI463227. B430203M17Rik. MGC183880. MMAC1. Pten. TEP1 |
| A_51_P266644 | Sesn1 | 0.63 | 1110002G11Rik. AU044290. MGC118148. Pa26. Sesn1. Sest1 |
| A_51_P461504 | Eef2k | 0.64 | C86191. Eef2k. eEF-2K |
| A_51_P268010 | Khdrbs1 | 0.64 | Khdrbs1. Sam68. p62. p68 |
| A_51_P515768 | Pdlim7 | 0.64 | 1110003B01Rik. 2410002J21Rik. AV007930. Enigma. Pdlim7 |
| A_52_P664656 | Acvr2a | 0.64 | ActrIIa. Acvr2. Acvr2a. TactrII |
| A_52_P351816 | Itgb1 | 0.64 | 4633401G24Rik. AA409975. AA960159. CD29. ENSMUSG00000051907. Fnrb. Gm9863. Itgb1. gpIIa |
| A_51_P237783 | Foxd1 | 0.64 | AI385632. BF-2. FREAC4. Foxd1. Hfh10. Hfhbf2 |
| A_52_P672496 | Plekho2 | 0.64 | AI840980. MGC30448. Plekho2. Plekhq1 |
| A_52_P199905 | Slc27a1 | 0.64 | FATP1. Fatp. Slc27a1 |
| A_52_P363068 | Nmnat2 | 0.64 | AI843915. D030041I09Rik. MGC113818. Nmnat2. PNAT1. PNAT2 |
| A_51_P428086 | Spnb2 | 0.64 | 9930031C03Rik. AL033301. KIAA4049. SPTB2. SPTBN1. Spnb-2. Spnb2. elf1. elf3. mKIAA4049 |
| A_51_P112932 | Entpd2 | 0.64 | Cd39l1. Entpd2. NTPDase2 |
| A_52_P140356 | Calm3 | 0.64 | CaMA. Calm1. Calm2. Calm3. R75142 |
| A_52_P474775 | Tspan32 | 0.64 | AW208513. Art-1. BB235973. D7Wsu37e. Phemx. Tspan32. Tssc6 |
| A_51_P163252 | Schip1 | 0.64 | Nf2ip. Schip-1. Schip1 |
| A_52_P560146 | Gm22 | 0.64 | Gm22 |
| A_51_P446232 | Dbn1 | 0.64 | Dbn1 |
| A_51_P279100 | Ptgs1 | 0.64 | COX1. Cox-1. Cox-3. Pghs1. Ptgs1 |
| A_52_P80806 | Syncrip | 0.64 | 2610109K23Rik. 4632417O19Rik. GRY-RBP. Nsap1. Nsap1l. Syncrip. pp68 |
| A_52_P449718 | Megf6 | 0.64 | 2600001P17Rik. Egfl3. MGC49785. Megf6 |
| A_51_P271704 | Cnn3 | 0.64 | 1600014M03Rik. C85854. Calpo3. Cnn3 |
| A_51_P306789 | 2810055G20Rik | 0.64 | 2810055G20Rik. D730043B02Rik |
| A_52_P432396 | Srcap | 0.64 | B930091H02Rik. D030022P06Rik. F630004O05Rik. Srcap |
| A_51_P506284 | Pdlim7 | 0.64 | 1110003B01Rik. 2410002J21Rik. AV007930. Enigma. Pdlim7 |
| A_52_P756306 | NA | 0.64 |  |
| A_52_P50090 | Srsf5 | 0.64 | MGC96781. Sfrs5. Srsf5 |
| A_52_P22446 | Dgcr8 | 0.64 | D16H22S1742E. D16H22S788E. D16Wis2. Dgcr8. Gy1. N41. Vo59c07 |
| A_52_P8587 | Cd109 | 0.64 | 9930012E15Rik. AI480638. Cd109. GARP |
| A_52_P180726 | Fam53a | 0.64 | 2410018C17Rik. 5430419M09Rik. Dntnp. Fam53a. KIAA4017 |
| A_51_P389988 | Slc40a1 | 0.64 | Dusg. Fpn1. IREG1. MTP. MTP1. Ol5. Pcm. Slc11a3. Slc39a1. Slc40a1 |
| A_51_P306067 | Lpp | 0.64 | 9430020K16Rik. AA959454. AU024130. B130055L10Rik. C79715. D630048H16. Lpp |
| A_52_P567200 | NA | 0.64 |  |
| A_51_P450373 | 2310068J16Rik | 0.64 | 2310068J16Rik |
| A_52_P643165 | Samd4 | 0.64 | 1700024G08Rik. 1700111L17Rik. 4933436G17Rik. Samd4. Samd4a. Smaug. Smaug1 |
| A_51_P475672 | Slc7a1 | 0.64 | 4831426K01Rik. AI447493. Atrc-1. Atrc1. Cat1. Rec-1. Rev-1. Slc7a1. mCAT-1 |
| A_52_P574306 | Apoc3 | 0.64 | Apoc. Apoc3 |
| A_52_P640152 | 2610005L07Rik | 0.64 | 2610005L07Rik. 2810038F24Rik. 6720476A01Rik |
| A_51_P416295 | Irgm2 | 0.64 | AI481100. GTPI. Iigp2. Irgm2. MGC102455 |
| A_51_P104977 | NA | 0.64 |  |
| A_51_P387123 | Oasl2 | 0.64 | M1204. Mmu-OASL. Oasl. Oasl2 |
| A_51_P254646 | Jdp2 | 0.64 | Jdp2. Jundm2. Jundp2. TIF |
| A_51_P166244 | Osbpl1a | 0.64 | G430090F17Rik. Gm753. Osbpl1a. Osbpl1b |
| A_51_P302204 | Cry1 | 0.64 | AU020726. AU021000. Cry1. Phll1 |
| A_52_P552062 | Fgfr1 | 0.64 | AW208770. FLG. Fgfr-1. Fgfr1. Flt-2. Hspy |
| A_52_P190506 | Mrpl15 | 0.64 | HSPC145. MRP-L7. Mrpl15. Rpml7 |
| A_52_P248595 | Otud6b | 0.64 | 2600013N14Rik. AU015433. MGC103361. Otud6b |
| A_52_P446644 | NA | 0.64 |  |
| A_51_P217682 | Pcf11 | 0.65 | 2500001H09Rik. 5730417B17Rik. C77803. KIAA0824. Pcf11 |
| A_52_P194289 | Iqsec1 | 0.65 | AW561907. BC026481. BRAG2. D6Ertd349e. Iqsec1. KIAA0763. cI-43. mKIAA0763 |
| A_51_P281380 | Tspan5 | 0.65 | 2810455A09Rik. 4930505M03Rik. AU024142. NET-4. Tm4sf9. Tspan5 |
| A_52_P661606 | Phgdh | 0.65 | 3-PGDH. 3PGDH. 4930479N23. A10. MGC113796. MGC117966. PGAD. PGD. PGDH. Phgdh. SERA |
| A_52_P315155 | Ephb2 | 0.65 | Cek5. Drt. ETECK. Ephb2. Erk. Hek5. Nuk. Prkm5. Qek5. Sek3. Tyro5 |
| A_51_P154840 | 1700080G18Rik | 0.65 | 1700080G18Rik |
| A_51_P332939 | Farp1 | 0.65 | AW228844. BC030329. Cdep. Farp1 |
| A_51_P141949 | B230120H23Rik | 0.65 | AV006891. B230120H23Rik. MLTK. MLTKalpha. MLTKbeta. Zak |
| A_51_P440682 | Cap1 | 0.65 | Cap1 |
| A_51_P307917 | Tmem179 | 0.65 | AI839735. Tmem179 |
| A_51_P439803 | Actg1 | 0.65 | AL023024. Actg. Actg1. Actl. E51. MGC117491. MGC28612 |
| A_52_P108607 | Dtna | 0.65 | 2210407P21Rik. A0. Dtn. Dtna. a-DB-1. adbn |
| A_51_P117477 | Slc27a1 | 0.65 | FATP1. Fatp. Slc27a1 |
| A_52_P119997 | Rhbdd1 | 0.65 | 4930418P06Rik. MGC7809. Rhbdd1 |
| A_51_P282760 | Per2 | 0.65 | Per2. mKIAA0347. mPer2 |
| A_52_P541826 | Eif4a1 | 0.65 | BM-010. Ddx2a. Eif4. Eif4a1 |
| A_52_P137371 | Hmgcr | 0.65 | HMG-CoAR. Hmgcr. MGC103269. Red |
| A_51_P150964 | Pdgfrb | 0.65 | AI528809. CD140b. Pdgfr. Pdgfrb |
| A_52_P234619 | Ptbp1 | 0.65 | AA407203. AL033359. HNRPI. PTB-1. PTB2. PTB3. PTB4. Ptb. Ptbp1. pPTB |
| A_51_P165504 | Twist2 | 0.65 | Dermo1. Twist2. bHLHa39 |
| A_52_P195602 | Cplx2 | 0.65 | 921-L. AI413745. AW492120. Cplx2 |
| A_52_P463183 | Asap1 | 0.65 | AV239055. Asap1. DEF-1. Ddef1. PAP. mKIAA1249.. s19 |
| A_52_P319265 | Sept6 | 0.65 | 2810035H17Rik. C920001C06Rik. KIAA0128. Sep6. Sept6. mKIAA0128 |
| A_52_P378975 | Slc1a3 | 0.65 | AI504299. B430115D02Rik. Eaat1. GLAST. GLAST-1. GLU-T. GluT-1. Gmt1. MGluT1. Slc1a3 |
| A_52_P72546 | 2810055G20Rik | 0.65 | 2810055G20Rik. D730043B02Rik |
| A_52_P92302 | Dnajb12 | 0.65 | Dj10. Dnajb12. mDj10 |
| A_51_P121547 | Bmi1 | 0.65 | AW546694. Bmi-1. Bmi1. Pcgf4 |
| A_51_P404815 | Apol6 | 0.65 | 2310076O14Rik. Apol6 |
| A_51_P300337 | Csrp2 | 0.65 | AW551867. Crp2. Csrp2. SmLim |
| A_51_P438805 | Txnip | 0.65 | 1200008J08Rik. AA682105. Hyplip1. THIF. Tbp-2. Txnip. VDUP1 |
| A_52_P422557 | NA | 0.65 |  |
| A_52_P479326 | Chpf2 | 0.65 | 2010209O12Rik. AW060945. Chpf2. mKIAA1402 |
| A_51_P464892 | 4930426L09Rik | 0.65 | 4930426L09Rik |
| A_52_P437421 | Bmper | 0.65 | 3110056H04Rik. Bmper. CV-2. Crim3. Cv2 |
| A_52_P191527 | Rnmt | 0.65 | 2610002P10Rik. AI848273. Rg7mt1. Rnmt. mKIAA0398 |
| A_52_P432124 | Gsta3 | 0.65 | Gst2-3. Gsta3 |
| A_51_P428345 | Mbnl1 | 0.65 | Mbnl. Mbnl1. mKIAA0428 |
| A_51_P429276 | Tmod3 | 0.65 | Tmod3. U-Tmod. UTMOD |
| A_52_P201206 | Scrn1 | 0.65 | 2810019K23Rik. 6330535A03Rik. AI852905. KIAA0193. SES1. Scrn1. mKIAA0193 |
| A_51_P428555 | Adh1 | 0.65 | ADH-AA. AI194826. Adh-1. Adh-1-t. Adh-1e. Adh-1t. Adh-3e. Adh1. Adh1-e. Adh1-t. Adh1tl. Adh3-e |
| A_52_P43848 | Sco1 | 0.65 | 2610001C07Rik. D11Bwg1310e. Sco1 |
| A_51_P270274 | Smc6 | 0.65 | 2810489L22Rik. 3830418C19Rik. AA990493. AU018782. AW742439. KIAA4103. MGC96146. SMC-6. Smc6. Smc6l1. mKIAA4103 |
| A_51_P427674 | Cpt1a | 0.66 | C730027G07. CPTI. Cpt1. Cpt1a |
| A_52_P139727 | Atf4 | 0.66 | Atf-4. Atf4. C/ATF. CREB2. MGC96460. TAXREB67 |
| A_52_P35801 | Marcksl1 | 0.66 | AL022768. AW215397. AW536807. D4Bc1. F52. MacMARCKS. Macs2. Macs3. Marcksl1. Mlp. Mrp |
| A_52_P649177 | Pdlim5 | 0.66 | 1110001A05Rik. AI987914. C87059. Enh. Enh1. Enh2. Enh3. LIM. Pdlim5 |
| A_51_P414481 | Tcerg1 | 0.66 | 2410022J09Rik. 2900090C16Rik. AI428505. CA150b. FBP23. FBP28. Taf2s. Tcerg1. ca150. p144 |
| A_51_P257550 | Marcksl1 | 0.66 | AL022768. AW215397. AW536807. D4Bc1. F52. MacMARCKS. Macs2. Macs3. Marcksl1. Mlp. Mrp |
| A_51_P156363 | Hnrnpul1 | 0.66 | E130317O14Rik. E1B-AP5. E1BAP5. Hnrnpul. Hnrnpul1. Hnrpul1. MGC36621. MGC78330 |
| A_51_P335758 | Chn1 | 0.66 | 0610007I19Rik. 0710001E19Rik. 1700112L09Rik. 2900046J01Rik. AI413815. ARHGAP2. Chn1 |
| A_51_P274173 | Ldlr | 0.66 | Hlb301. Ldlr |
| A_51_P291438 | Tnc | 0.66 | AI528729. C130033P17Rik. Hxb. MGC144208. MGC144209. TN. TN-C. Ten. Tnc. cytotactin. tenascin-C |
| A_52_P229044 | Slc20a2 | 0.66 | MolPit2. Pit-2. Pit2. Ram-1. Ram1. Slc20a2 |
| A_51_P103912 | Mc5r | 0.66 | Mc5r |
| A_52_P589169 | 2010111I01Rik | 0.66 | 2010111I01Rik. 2300006M17Rik. AP-O. Aopep |
| A_51_P137236 | Olfm1 | 0.66 | AMY. AW742568. Noe1. OlfA. Olfm1. Pancortin. Pancortin3 |
| A_51_P327796 | Itgb5 | 0.66 | AA475909. AI874634. ESTM23. Itgb5. [b]-5. [b]5. [b]5A. [b]5B. beta-5. beta5 |
| A_52_P536938 | Kif21a | 1.52 | AI850764. Kif21a. mKIAA1708 |
| A_52_P663585 | Sept10 | 1.53 | 4921515A04Rik. 9430099J10Rik. AA408298. AI874685. Sept10 |
| A_52_P467096 | Golga4 | 1.53 | AI225887. AU019508. Golga4. Olp-1 |
| A_52_P225331 | Dusp19 | 1.53 | 5930436K22Rik. C79103. Dusp19. SKRP1. TS-DSP1 |
| A_52_P513477 | NA | 1.54 |  |
| A_51_P206585 | Runx1 | 1.55 | AI462102. AML1. Cbfa2. Pebp2a2. Pebpa2b. Runx1 |
| A_52_P287058 | Ufm1 | 1.55 | 1810045K17Rik. AI132708. AI463323. ENSMUSG00000074598. Gm10726. Ufm1 |
| A_51_P423484 | Rbp1 | 1.55 | CRBPI. Crbp. Rbp-1. Rbp1 |
| A_51_P196629 | NA | 1.56 |  |
| A_51_P518600 | Atp6v1c2 | 1.56 | 1110038G14Rik. Atp6v1c2 |
| A_51_P387913 | Cox17 | 1.57 | AI037035. Cox17 |
| A_51_P157154 | Grin2d | 1.57 | GluN2D. Grin2d. NMDAR2D. NR2D |
| A_51_P145735 | Acyp1 | 1.57 | 1110039O14Rik. AI325944. Acyp1 |
| A_51_P394946 | Dusp19 | 1.57 | 5930436K22Rik. C79103. Dusp19. SKRP1. TS-DSP1 |
| A_52_P321318 | NA | 1.58 |  |
| A_52_P368532 | Spry2 | 1.59 | Spry2. sprouty2 |
| A_51_P138933 | Ppp1r3b | 1.6 | 6430576E21. AW821953. GL. Ppp1r3b |
| A_52_P336748 | NA | 1.6 |  |
| A_51_P295192 | Nfkbia | 1.6 | AI462015. Nfkbi. Nfkbia |
| A_51_P317505 | Nat1 | 1.61 | Nat-1. Nat1 |
| A_51_P431433 | Tmem2 | 1.61 | 3110012M15Rik. MGC102198. Tmem2. mKIAA1412 |
| A_51_P179647 | NA | 1.61 |  |
| A_51_P469411 | Traf3 | 1.62 | AI528849. CAP-1. CD40bp. CRAF1. LAP1. T-BAM. Traf3. amn |
| A_52_P99888 | Cxcl16 | 1.62 | 0910001K24Rik. AV290116. BB024863. CXCL16v1. CXCL16v2. Cxcl16. SR-PSOX. Zmynd15 |
| A_52_P597860 | Wasf2 | 1.65 | AW742646. D4Ertd13e. WAVE2. Wasf2 |
| A_52_P573552 | Trib1 | 1.65 | A530090O15Rik. TRB-1. Trb1. Trib1 |
| A_51_P383991 | Sept4 | 1.67 | Bh5. Gm11492. OTTMUSG00000001265. Pnutl2. RP23-168C20.1. Sept4 |
| A_51_P201390 | NA | 1.69 |  |
| A_51_P122246 | Creld2 | 1.71 | 5730592L21Rik. C85758. Creld2 |
| A_51_P159453 | Serpina3n | 1.72 | Serpina3n. Spi2-2. Spi2.2. Spi2/eb.4 |
| A_51_P265571 | Adm | 1.72 | AM. Adm |
| A_52_P533280 | Prm1 | 1.73 | Prm-1. Prm1 |
| A_52_P1020860 | AW112010 | 1.73 | AW112010 |
| A_52_P510592 | Adamts2 | 1.75 | 4732482M10. A430089F14. ADAM-TS2. ADAMTS-3. Adamts2. KIAA4060. PCINP. hPCPNI. mKIAA4060 |
| A_51_P440238 | Ggt6 | 1.76 | 9030405D14Rik. AI427679. AW490214. Ggt6 |
| A_51_P346938 | Lrg1 | 1.76 | 1300008B03Rik. 2310031E04Rik. Lrg. Lrg1. Lrhg. MGC102387 |
| A_52_P363216 | Gcnt2 | 1.77 | 5330430K10Rik. Gcnt2. IGnT. IGnTA. IGnTB. IGnTC |
| A_52_P556111 | 4933426M11Rik | 1.77 | 4933426M11Rik. mKIAA0247 |
| A_52_P551851 | Tspan11 | 1.77 | 1110014F12Rik. AU067806. Tspan11 |
| A_51_P499254 | Obfc2a | 1.8 | 4930434H03Rik. 4930442A21Rik. 4930488J04Rik. 4933440J18Rik. 5830411E10Rik. AI852561. Nabp1. Nbp1. Obfc2a |
| A_51_P185660 | Ccl9 | 1.82 | CCF18. Ccl9. MRP-2. Scya10. Scya9 |
| A_52_P484903 | Fosl2 | 1.84 | Fosl2. Fra-2 |
| A_51_P326826 | NA | 1.85 |  |
| A_51_P389751 | Relb | 1.86 | MGC143683. MGC143684. Relb |
| A_51_P389265 | Pnpla3 | 1.86 | Adpn. Pnpla3 |
| A_52_P666646 | 4933426M11Rik | 1.87 | 4933426M11Rik. mKIAA0247 |
| A_51_P459661 | Lipa | 1.88 | AA960673. Lal. Lip-1. Lip1. Lipa |
| A_51_P128876 | Ifitm3 | 1.89 | 1110004C05Rik. Cd225. Cdw217. Fgls. IP15. Ifitm3. mil-1 |
| A_51_P115575 | Map3k8 | 1.91 | Cot. Cot/Tpl2. Est. Estf. Map3k8. Tpl-2. Tpl2. c-COT |
| A_52_P748882 | Eno2 | 1.95 | AI837106. D6Ertd375e. Eno-2. Eno2. NSE |
| A_51_P338443 | Angptl4 | 1.96 | Angptl4. Arp4. Bk89. Fiaf. Hfarp. Ng27. Pgar. Pgarg. Pp1158 |
| A_51_P195958 | Phlda1 | 2 | DT1P1B11. Phlda1. TDAG51. Tdag |
| A_51_P317176 | Csf3 | 2.01 | Csf3. Csfg. G-CSF. MGI-IG |
| A_51_P481342 | Ugcg | 2.01 | AU043821. C80537. Epcs21. GlcT-1. Ugcg. Ugcgl |
| A_51_P271503 | Il1r1 | 2.04 | CD121a. CD121b. IL-iR. Il1r-1. Il1r1. MGC129154 |
| A_51_P484158 | Steap1 | 2.05 | 2410007B19Rik. Prss24. Steap. Steap1 |
| A_51_P319460 | Osmr | 2.06 | OSMRB. Osmr |
| A_51_P502614 | Dusp6 | 2.08 | 1300019I03Rik. Dusp6. MGC98540. MKP-3. MKP3. PYST1 |
| A_52_P678373 | Gm12830 | 2.09 | Gm12830. OTTMUSG00000008584 |
| A_51_P480119 | Prelid2 | 2.1 | 1700003A01Rik. C330008K14Rik. Prelid2 |
| A_51_P337944 | Bmp2k | 2.1 | 4933417M22Rik. AA673486. AV128808. BIKE. Bmp2k |
| A_51_P332717 | Ugcg | 2.11 | AU043821. C80537. Epcs21. GlcT-1. Ugcg. Ugcgl |
| A_52_P184257 | Steap1 | 2.12 | 2410007B19Rik. Prss24. Steap. Steap1 |
| A_52_P654841 | Plscr1 | 2.15 | MmTRA1a. MmTRA1b. Nor1. Plscr1. Tra1. Tra1a. Tra1b. Tras1. Tras2 |
| A_51_P111164 | Rnd1 | 2.16 | A830014L09Rik. Arhs. Rnd1 |
| A_52_P552194 | Il13ra1 | 2.18 | AI882074. CD213a1. IL-13r[a]. Il13ra. Il13ra1. NR4 |
| A_52_P502754 | Ampd3 | 2.2 | Ampd3 |
| A_52_P567281 | NA | 2.21 |  |
| A_51_P338924 | Ifnar2 | 2.21 | AI747302. Ifnar-2. Ifnar2 |
| A_51_P308796 | Fosl1 | 2.26 | AW538199. Fosl1. Fra1. fra-1 |
| A_51_P345393 | Fas | 2.27 | AI196731. APO1. APT1. CD95. Fas. TNFR6. Tnfrsf6. lpr |
| A_51_P454008 | Lbp | 2.29 | Bpifd2. Lbp. Ly88 |
| A_51_P172853 | Cd14 | 2.33 | Cd14 |
| A_51_P173678 | Slc10a6 | 2.33 | 8430417G17Rik. C78479. MGC149979. Slc10a6. Soat |
| A_52_P87713 | Timp1 | 2.38 | Clgi. MGC7143. TIMP-1. Timp. Timp1 |
| A_51_P120830 | Mmp10 | 2.43 | AV377895. MMP-10. Mmp10. SL-2 |
| A_51_P202408 | Ptgir | 2.45 | IP. PGI2. Ptgir |
| A_52_P481957 | Grem1 | 2.5 | Cktsf1b1. Drm. Grem1. ld |
| A_52_P419455 | Adora2b | 2.53 | A2BAR. A2BR. A2b. AA2BR. AI480866. AI605384. Adora2b. MGC144574. MGC144575 |
| A_51_P444447 | Cebpd | 2.57 | Cebpd |
| A_51_P512379 | NA | 2.57 |  |
| A_51_P171215 | Ereg | 2.58 | EPR. Ereg. MGC36144 |
| A_52_P1093529 | Pik3r5 | 2.59 | AV230647. F730038I15Rik. Pik3r5 |
| A_51_P338803 | Pigt | 2.59 | 2510012P17Rik. 4930534E15Rik. CGI-06. NDAP. Ndap7. Pigt |
| A_51_P210956 | Vcam1 | 2.6 | CD106. Vcam-1. Vcam1 |
| A_51_P159201 | Junb | 2.6 | Junb |
| A_51_P511787 | Casp4 | 2.63 | Casp11. Casp4. Caspl. ich-3 |
| A_52_P311031 | NA | 2.65 |  |
| A_51_P449171 | Birc3 | 2.66 | AW107670. Api1. Api2. Birc2. Birc3. HIAP2. IAP1. IAP2. MIAP1. MIAP2. MIHB. MIHC. RNF49. cIAP-1. cIAP-2. cIAP1. cIAP2 |
| A_51_P159194 | Junb | 2.67 | Junb |
| A_51_P365189 | Il13ra1 | 2.7 | AI882074. CD213a1. IL-13r[a]. Il13ra. Il13ra1. NR4 |
| A_52_P412417 | Tnfaip3 | 2.72 | A20. Tnfaip3. Tnfip3 |
| A_51_P249989 | Tifa | 2.76 | T2bp. Tifa |
| A_52_P444628 | Cybb | 2.79 | C88302. Cgd. Cybb. Cyd. Nox2. gp91phox |
| A_51_P229676 | Plscr1 | 2.84 | MmTRA1a. MmTRA1b. Nor1. Plscr1. Tra1. Tra1a. Tra1b. Tras1. Tras2 |
| A_52_P662562 | NA | 2.85 |  |
| A_51_P286488 | Ier3 | 2.89 | AI663993. IEX-1. Ier3. cI-3. gly96 |
| A_52_P93910 | Nrp2 | 2.91 | 1110048P06Rik. Np-2. Np2. Npn-2. Npn2. Nrp2 |
| A_51_P282983 | Slc39a14 | 3.05 | BC021530. G630015O18Rik. MGC38539. Slc39a14. Zip14. fad123 |
| A_52_P319438 | Ankrd37 | 3.06 | Ankrd37 |
| A_51_P297925 | Zc3h12a | 3.06 | BC036563. MCPIP. MGC41320. Mcpip1. Zc3h12a |
| A_51_P512384 | Timp1 | 3.07 | Clgi. MGC7143. TIMP-1. Timp. Timp1 |
| A_51_P229675 | Plscr1 | 3.12 | MmTRA1a. MmTRA1b. Nor1. Plscr1. Tra1. Tra1a. Tra1b. Tras1. Tras2 |
| A_51_P374726 | Ptx3 | 3.13 | AI607804. Ptx3. TSG-14 |
| A_51_P255699 | Mmp3 | 3.21 | Mmp3. SLN-1. SLN1. STR-1. Stmy1. Str1 |
| A_52_P26161 | Ptx3 | 3.32 | AI607804. Ptx3. TSG-14 |
| A_51_P184484 | Mmp13 | 3.54 | Clg. MMP-13. Mmp1. Mmp13 |
| A_51_P452629 | Tlr2 | 3.58 | Ly105. Tlr2 |
| A_52_P503663 | O3far1 | 3.58 | AI552415. GT01. Gpr120. KPG_013. O3far1 |
| A_51_P458258 | Ccl19 | 3.86 | CKb11. Ccl19. ELC. MIP3B. Scya19. exodus-3 |
| A_51_P387591 | Nfkbiz | 3.9 | AA408868. INAP. Mail. Nfkbiz |
| A_51_P254855 | Ptgs2 | 3.93 | COX2. Cox-2. PGHS-2. PHS-2. Pghs2. Ptgs2. TIS10 |
| A_52_P639522 | Serpinb2 | 3.98 | PAI-2. Planh2. Serpinb2. ovalbumin |
| A_51_P374203 | Cxcl16 | 4.12 | 0910001K24Rik. AV290116. BB024863. CXCL16v1. CXCL16v2. Cxcl16. SR-PSOX. Zmynd15 |
| A_52_P700056 | Gm11428 | 4.15 | Amwap. Gm11428. OTTMUSG00000000971 |
| A_51_P314501 | Lars2 | 4.52 | AI035546. Kiaa0028. LEURS. Lars2 |
| A_51_P361650 | Saa1 | 4.53 | Saa-1. Saa1. Saa2 |
| A_51_P136542 | Bcl3 | 4.63 | AI528691. Bcl-3. Bcl3 |
| A_51_P166886 | Saa2 | 4.71 | AW111173. Saa-2. Saa1. Saa2 |
| A_51_P235123 | Nfkbie | 5.22 | IKBE. Nfkbie |
| A_52_P472324 | Slpi | 5.82 | Slpi |
| A_52_P419679 | Serpina3f | 6.91 | 2A1. BC049975. Serpina3f |
| A_51_P217218 | Il6 | 6.97 | Il-6. Il6 |
| A_51_P436652 | Ccl7 | 7.07 | Ccl7. MCP-3. Scya7. fic. marc. mcp3 |
| A_52_P419678 | Serpina3f | 7.08 | 2A1. BC049975. Serpina3f |
| A_52_P318673 | Saa1 | 7.54 | Saa-1. Saa1. Saa2 |
| A_52_P208763 | Ccl7 | 7.66 | Ccl7. MCP-3. Scya7. fic. marc. mcp3 |
| A_51_P326191 | Serpina3g | 11.18 | 2A2. AI119734. MGC107057. Serpina3g. Spi2-1. Spi2/eb.1. Spi2A. spi2 |
| A_51_P286737 | Ccl2 | 14.31 | AI323594. Ccl2. HC11. JE. MCAF. MCP-1. MCP1. SMC-CF. Scya2. Sigje |
| A_51_P337308 | Saa3 | 40.72 | AV098916. Saa-3. Saa3. l7R3 |
| A_51_P363187 | Cxcl1 | 47.23 | Cxcl1. Fsp. Gro1. KC. Mgsa. N51. Scyb1. gro |
| A_52_P295432 | Cxcl5 | 105.5 | AMCF-II. Cxcl5. ENA-78. GCP-2. LIX. Scyb5. Scyb6 |
